# Immunometabolism in obesity: Understanding the beneficial and detrimental roles of inflammation

**DOI:** 10.1371/journal.pbio.3003620

**Published:** 2026-01-21

**Authors:** Yun Sok Lee

**Affiliations:** Department of Medicine, Division of Endocrinology and Metabolism, University of California San Diego, La Jolla, California, United States of America; University of Birmingham, UNITED KINGDOM OF GREAT BRITAIN AND NORTHERN IRELAND

## Abstract

In obesity, nutrient excess and altered adipocyte secretory profiles reprogram cell-intrinsic metabolism, leading to the activation of immune cells within metabolically active tissues such as adipose tissue. This obesity-associated chronic low-grade metabolic inflammation (often referred to as metaflammation) is a well-established driver of insulin resistance and metabolic dysfunction. However, several lines of emerging evidence suggest that metaflammation is not merely a pathologic process, but may also serve as an adaptive response that supports metabolic homeostasis, particularly at the early stages of obesity. This Essay discusses immunometabolic mechanisms underlying the dual nature of metaflammation in obesity, highlighting how its initially beneficial effects can transition into detrimental outcomes.

## Abstract 2

Chronic low-grade metabolic inflammation (known as metaflammation) drives insulin resistance and metabolic dysfunction in obesity. This Essay discusses the immunometabolic mechanisms underlying metaflammation with a focus on its lesser known beneficial effects and how they can transition into detrimental outcomes.

## Introduction

The incidence of obesity is rapidly increasing across the globe and has now reached epidemic proportions [1]. Obesity is a major risk factor for metabolic syndrome, a cluster of conditions that increase the risk of cardiovascular disease and type 2 diabetes mellitus (T2D). Therefore, the ongoing global obesity epidemic can be seen as the primary driver behind the increasing prevalence of metabolic disorders [2]. Although several pathways have been proposed to explain how obesity contributes to the development of metabolic syndrome, the underlying mechanisms remain incompletely understood. A critical factor in this link is insulin resistance. Obesity is the most prevalent cause of insulin resistance in humans. As insulin resistance develops, the pancreatic beta cells typically compensate by increasing insulin secretion to maintain blood glucose levels within a normal range. However, when beta cells can no longer sustain hyperinsulinemia, blood glucose levels are persistently increased (i.e., in T2D). Multiple mechanisms have been proposed for the cause of obesity-associated insulin resistance and beta cell dysfunction [3–12]. In this Essay, I focus on chronic metabolic inflammation (often referred to as metaflammation), with a particular focus on the mechanisms by which obesity triggers metaflammation and how these processes subsequently influence insulin sensitivity and glucose and lipid metabolism, processes that are collectively encompassed by the concept of immunometabolism [13].

One of the seminal studies suggesting a direct effect of chronic inflammation in glycemic control was conducted by Grunfeld and Feingold. Their research demonstrated that administration of TNF induces hyperglycemia without altering plasma insulin levels in rodents [14]. The connection between obesity-induced insulin resistance and inflammation was first suggested in other early landmark studies by Hotamisligil and Spiegelman, showing that TNF levels are elevated in the adipose tissue of obese mice and that neutralizing TNF ameliorates insulin resistance [15,16]. These findings established a foundational understanding that inflammation can directly contribute to the development of insulin resistance and impaired glycemic control in obesity. In 2003, two studies published concurrently demonstrated that obesity-induced inflammation is associated with increased macrophage accumulation in adipose tissue [17,18]. This was seen in both mouse and human adipose tissue. To date, numerous studies have revealed that chronic obesity is associated with increased levels of various immune cell types (including macrophages), cytokines, chemokines (such as CCL2/MCP-1 and IL-1β), as well as other molecules within proximal pro-inflammatory signaling pathways (including JNK and IKKβ). Chronic increases in these pro-inflammatory mediators can impair insulin signaling in classical insulin target tissues, such as the liver, skeletal muscle, and adipose tissue [13,19–26]. While these concepts are largely derived from studies in rodents, studies in human adipose tissue [27–29], liver [30,31], primary adipocytes [32], skeletal muscle [33], pancreatic islets [34], and organoids [35] also support these concepts [36]. Plasma levels of pro-inflammatory cytokines, chemokines, and other related soluble factors (such as TNF, CCL2, IL-6, and PAI-1) are elevated in obese individuals compared with lean individuals. Levels of some of these factors, in particular PAI-1, can predict the severity of insulin resistance and the development of metabolic dysfunction-associated steatotic liver disease (MASLD) among obese individuals [37–39]. Intriguingly, however, emerging evidence also suggests that acute inflammatory responses may paradoxically support metabolic adaptation during the early stages of obesity. This Essay explores these seemingly dichotomous facets of metaflammation.

## The dual facets of metaflammation

Because inflammatory responses are detected at an early stage during the development of obesity and are typically associated with insulin resistance in obese individuals and in animal models of obesity, previous studies have mostly focused on how inflammation affects insulin resistance and metabolic dysfunction [13,40,41]. The proposed mechanisms are largely associated with proximal pro-inflammatory pathways, including TNF, IL-1β, and the CCL2–CCR2 system. However, clinical studies of agents targeting these pathways have raised safety concerns and shown disappointing efficacy, although some showed positive metabolic effects [19,42–47]. Indeed, inflammation is a physiological process necessary for defending the body against infection and tissue injury and for promoting tissue repair [48]. Moreover, although inflammation contributes to the development of insulin resistance and glucose intolerance in the context of chronic obesity, at the early stages of obesity, inflammation is not necessary for the development of these metabolic abnormalities [49,50]. For example, while depletion of macrophages, loss of lymphocytes (via Rag1 deficiency), or inhibition of pro-inflammatory immune cell activation (via hematopoietic cell-specific JNK deficiency) all protect mice from insulin resistance and glucose intolerance in the context of chronic obesity, these same strategies do not prevent the development of insulin resistance and glucose intolerance in mice fed a short term 1 week) high-fat diet (HFD) [49]. Furthermore, in humans, acute weight gain from one week of high-calorie intake leads to the development of insulin resistance without inflammation [50]. Instead, evidence indicates that certain acute inflammatory pathways activated at an early stage during the development of obesity contribute to maintaining vascular density and integrity, regulating local iron levels and lipolysis, clearing extracellular lipids and dead cell debris, supporting adipose tissue expandability, healthy remodeling of adipose tissue, and safe storage of excess lipids [51–55]. For example, although inhibiting TNF signaling using a neutralizing antibody after the onset of obesity improves metabolic abnormalities (at least, in rodents) [15], blocking early TNF or NF-κB signaling in adipocytes restricts adipose tissue expansion (by reducing adipogenesis) during HFD and exacerbates adipocyte hypertrophy, liver steatosis, glucose, and insulin intolerance in mice [54,56]. Likewise, adipocyte-specific depletion of TLR4 worsens insulin resistance and glucose intolerance in obese mice [55], although global or hematopoietic cell-specific TLR4 depletion, or global depletion of a TLR4 coreceptor CD14, ameliorates adipose tissue inflammation and insulin resistance in chronically obese mice [57,58–61]. These results suggest that while metaflammation generally has a key role in the development of insulin resistance in chronic established obesity, it is dispensable for triggering insulin resistance and metabolic dysfunction in the early stages of obesity. And specific inflammatory pathways (particularly within adipocytes) support the maintenance of metabolic homeostasis [62].

Similar to this concept, the well-known pro-inflammatory IκB kinase β (IKKβ) signaling pathway not only induces insulin resistance and metabolic dysfunction, but can also contribute to metabolic homeostasis, depending on when and where it is activated. For example, systemic haplodeficiency of *Ikbkb* (the gene encoding IKKβ) protects from the development of insulin resistance and glucose intolerance in obese mice [63]. Moreover, global IKKβ deficiency prevents the onset of muscle insulin resistance in mice infused with lipids [64], indicating that IKKβ is necessary for the development of insulin resistance and glucose intolerance induced by both acute lipid exposure and chronic obesity. Tissue-specific genetic manipulations have delineated the compartmental contributions of IKKβ: myeloid lineage-specific IKKβ depletion attenuates systemic inflammation and ameliorates insulin resistance in obese mice, whereas hepatocyte-specific IKKβ depletion or IKKβ inactivation (via hepatocyte-specific IκB overexpression) selectively improves liver-specific insulin sensitivity [65]. Conversely, hepatocyte-specific overexpression of constitutively active IKKβ impairs hepatic insulin action in lean mice [66], indicating that (sub)chronic baseline IKKβ hyperactivity alone is sufficient to cause hepatic insulin resistance. Notably, however, physiological post-prandial activation of hepatocyte IKKβ phosphorylates and activates XBP1, thereby facilitating adaptive responses to endoplasmic reticulum (ER) stress and maintaining metabolic homeostasis. In obesity, this beneficial hepatic IKKβ–XBP1 axis is attenuated. Genetic manipulation to further increase hepatocyte-specific IKKβ in the liver of obese mice alleviates ER stress and improves glucose metabolism [67]. Furthermore, in adipocytes, IKKβ enhances adipose tissue inflammation, but improves (not worsens) insulin sensitivity in mice fed a HFD by promoting the production of the anti-inflammatory cytokine IL-13 [68]. Therefore, it appears that precise spatiotemporal regulation of IKKβ pro-inflammatory activity is essential for maintaining metabolic homeostasis.

These results suggest that not all facets of metaflammation are pathogenic and that its metabolic impact depends critically on when, where, and how metaflammation occurs, affecting specific tissue functions rather than individual factors that exert a uniform systemic effect. Consistent with this view, although the expression of most pro-inflammatory cytokines is elevated in obese versus lean individuals and they are biologically active within induced tissue sites (e.g., adipose tissue), their systemic plasma concentrations (with the exception of PAI-1) are comparable between metabolically healthy and unhealthy obesity and often remain below the levels required to elicit insulin resistance [69].

It is interesting to note that adipose tissue inflammation is induced not only in the context of obesity but also during weight loss. For example, prolonged calorie restriction and weight loss increase the number of adipose tissue macrophages (ATMs) in nonobese individuals [70,71]. Similarly, the abundance of ATMs displays a transient increase within 3–7 days following weight loss in mice maintained on normal chow diet (lean) or HFD (obese) regimens [71], which gradually declines over time and is eventually normalized [72]. However, this acute increase in the number of ATMs during weight loss does not raise pro-inflammatory cytokine expression in ATMs or adipose tissue [72]. Instead, it accompanies increased lipid uptake by ATMs and upregulates lipid metabolism genes in adipocytes [71]. Depletion of macrophages using clodronate liposomes enhances fasting-induced adipose tissue lipolysis and raises plasma free fatty acid (FFA) levels, suggesting that ATMs are necessary for tempering excess lipid release from adipose tissue during weight loss. These observations align with the perspective that inflammation serves as an adaptive response rather than solely as a pathological process, and also highlights the importance of qualitative rather than merely quantitative alterations in ATM abundance and other inflammation metrics. Probably due to this inherent complexity, although weight loss eventually leads to improvements in metaflammation, depending on the experimental timeline, it is often observed that the numbers of ATMs remain elevated for weeks during weight loss, even when metabolic improvements (e.g., glucose tolerance and insulin sensitivity) manifest [37,73–76]. This is often cited as evidence for questioning the causal role of inflammation in obesity-induced metabolic dysfunction [77,78]. However, specific characteristics of inflammation (including the predominant ATM subtypes and their attendant gene expression profiles) induced during weight gain (obesity) versus weight loss may substantially diverge between these states [79]. Therefore, it is likely that metaflammation is not inherently pathological, but rather its impact on metabolic health is dependent upon contextual determinants, the acuity versus chronicity of the inflammatory response, and the specific constituents of the inflammatory milieu. Delineating the molecular determinants governing the transition from protective to detrimental metaflammation during the development of obesity will be imperative in the future.

## Initiation of metaflammation

Metaflammation is characterized by distinct immune cell composition, cytokine profiles, and histologic features compared with classical infection-induced inflammation, despite sharing some common features [13]. Although unique molecular signatures continue to be identified, one of the most widely used indicators of metaflammation is increased expression of pro-inflammatory cytokines and chemokines, accompanied by the accumulation of macrophages in metabolically active tissues, particularly adipose tissue. When these parameters are used to assess metaflammation during the development of obesity, increased expression of pro-inflammatory cytokines and infiltration of macrophages can be detected in visceral adipose tissue (VAT) as early as three days after starting a HFD [49,80–82]. This occurs before other peripheral tissues, including subcutaneous adipose tissue (SAT), liver, and skeletal muscle, show signs of inflammation. As obesity develops, VAT inflammation intensifies, paralleling a progressive decline in insulin sensitivity and glucose tolerance [49]. For example, in lean/healthy mice, macrophages constitute approximately 5%–10% of stromal vascular cells in VAT. After 7 days on a HFD, the proportion of ATMs is increased by 15%−25%, accompanied by a 34% decrease in systemic insulin sensitivity, as measured by glucose infusion rate during hyperinsulinemic-euglycemic clamp studies. This trend continues with prolonged HFD and, once obesity becomes fully established, these changes tend to plateau with ATMs making up as much as 40% of all stromal vascular cells, and systemic insulin sensitivity declining by as much as 77% [49]. Inhibition of adipose tissue inflammation through targeted depletion of adipocyte-specific JNK, HIF-1α, or CCL2 (which promotes immune cell infiltration into adipose tissue and activation) improves insulin resistance in adipose tissue, liver, and skeletal muscle and mitigates glucose intolerance in obese mice [83–88], highlighting the primacy of adipose tissue inflammation in systemic insulin action and glycemic control in chronic obesity.

**Fig 1:**
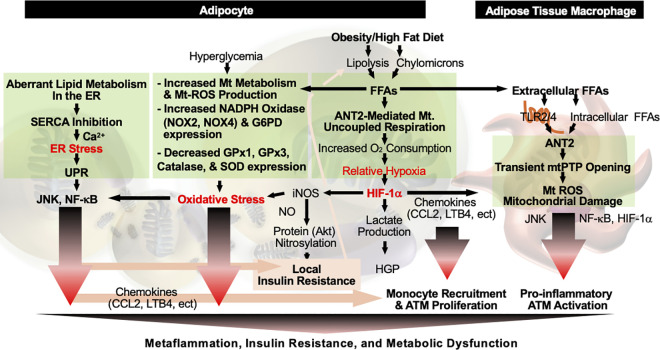
Initiation of metaflammation in adipose tissue. Early during the development of obesity, metaflammation first emerges in visceral adipose tissue as an adaptive response to increased oxygen demand, relative hypoxia, and metabolic stress (e.g., ER stress and oxidative stress). While these changes primarily accumulate within adipocytes, prolonged nutrient excess and resulting alterations in adipocyte secretory profiles promote changes in immune cell populations (including macrophages), thereby amplifying the inflammatory response.

The mechanism by which obesity induces adipose tissue inflammation involves the accumulation of cellular stress (such as metabolic stress, oxidative stress, or hypoxia) and the activation of stress response pathways (such as increased expression of HIF-1α and unfolded protein response; Fig 1). As the earliest change that can trigger metaflammation, adipose tissue hypoxia can be detected as early as 1 day after starting a HFD. The decrease in oxygen tension occurs selectively in adipose tissue and is not observed in skeletal muscle (when measured at a sedentary condition), kidney, or the liver (albeit the liver develops a relatively moderate hypoxia at the later stages of obesity) [89–91]. At the molecular level, elevated levels of FFAs (from either chylomicrons or increased lipolysis) induce an ANT2-dependent increase in mitochondrial oxygen consumption in adipocytes. In addition, as adipose tissue expands, its growth outpaces the development of new blood vessels (vascularization) required to deliver sufficient oxygen [62,92–96]. Furthermore, increased infiltration of immune cells and (myo)fibroblasts in chronic obesity can further raise adipose tissue oxygen demand. These changes collectively lead to the decreases in adipose tissue oxygen tension (hypoxia) and a subsequent increase in HIF-1α expression [84,90]. HIF-1α induces the expression of genes that facilitate adaptation to low oxygen concentration, including those involved in anaerobic metabolism (e.g., *Slc2a1* and *Pdk1*) and angiogenesis (e.g., *Vegf* and various chemokines). While acute increases in VEGF, pro-inflammatory cytokines (IL-6), chemokines (CCL2), and lipokines (leukotriene B4) mediated by HIF-1α promote endothelial cell recruitment and angiogenesis, chronic increases therein drive the recruitment of circulating monocytes, which differentiate into pro-inflammatory ATMs [25,84,87,97,98]. Moreover, CCL2 promotes ATM proliferation [99]. HIF-1α further elicits fibrogenic gene expression, leading to adipose tissue fibrosis and endotrophin production, which in turn amplifies inflammation [88,100–102]. Concomitantly, HIF-1α upregulates *Nos2, Slc2a1,* and *Pdk1*, thereby augmenting nitric oxide (NO) and lactate production. Elevated NO levels induce S-nitrosylation of critical insulin signaling components, impairing their functionality and contributing to insulin resistance in adipose tissue. Furthermore, lactate generated by hypoxic adipocytes in obesity is released into the systemic circulation, where it serves as a substrate for hepatic gluconeogenesis, ultimately contributing to hyperglycemia. Adipocyte-specific overexpression of a constitutively active form of HIF-1α is sufficient to induce adipose tissue inflammation and fibrosis in mice fed a normal chow diet [103]. Conversely, pharmacological inhibition of HIF-1α, genetic deletion of adipocyte-specific *Hif1a*, or adipocyte-specific overexpression of a dominant-negative form of HIF-1α ameliorates adipose tissue inflammation, as well as insulin resistance and glucose tolerance, in obese mice [84–86,88,104]. These effects are predominantly mediated by HIF-1α, but not HIF-2α [84,105–107], although HIF-1α shares structural similarities and several common target genes with HIF-2α. Taken together, these results suggest that adipose tissue hypoxia and HIF-1α have a central role in triggering metaflammation, and that metaflammation begins as an adaptive response to increased oxygen demand and metabolic stress, although it eventually leads to metabolic dysfunction.

## Expansion and propagation of metaflammation

Adipose tissue hypoxia persists throughout chronic obesity in both mice [84,90,91,101,108,109] and humans [90,110–113] (although some studies in humans have reported contradictory findings [114,115]). Adipose tissue oxygenation correlates with insulin sensitivity in obese individuals [113]. Moreover, as obesity progresses in a (sub)chronic manner, sustained nutrient excess disrupts lipid metabolism, increasing the phosphatidylcholine/phosphatidylethanolamine ratio in the ER membrane [116–118]. This imbalance impairs sarco/ER calcium ATPase function, resulting in ER Ca^2+^ depletion and defective protein folding, thereby triggering ER stress in adipocytes and hepatocytes. Additionally, prolonged nutrient overload elevates mitochondrial reactive oxygen species (ROS) production and induces cytosolic pro-oxidant enzymes (e.g., *Nox4, Nos2*, and *G6pd*), while simultaneously downregulating antioxidant counterparts (*Sod1, GPx1, GPx3,* and *Cat*) in adipose tissue [119–122], resulting in enhanced oxidative stress. While hypoxia, ER stress, and oxidative stress likely accumulate primarily in metabolically active cell types, including adipocytes and hepatocytes, and initiate inflammatory responses, prolonged nutrient excess and soluble factors released from hypoxic/stressed adipocytes (e.g., altered adipocytokine and exosome secretion profiles and increased FFAs and ceramide release) also trigger alterations in the number (growth and trafficking) and function of various immune cells, including macrophages, neutrophils, eosinophils, natural killer (NK) cells, regulatory T (T_reg_) cells, natural killer T (NKT) cells, CD8^+^ T cells, and B cells, thereby contributing to the amplification of inflammation [13,40,41,48]. Changes in the secretory profiles of adipocytes, hepatocytes, and infiltrating immune cells collectively disrupt both inter-organ and intra-organ communication by altering paracrine and endocrine signaling, as well as sympathetic innervation, ultimately leading to adipose tissue dysfunction, systemic insulin resistance, and metabolic dysfunction [123]. For example, FFAs activate the TLR4 and ANT2/HIF-1α pathways within ATMs, leading to pro-inflammatory ATM activation [124–126]. Additionally, factors released from hypoxic adipocytes in obesity (e.g., increased leptin and FFAs and decreased adiponectin levels in the plasma) promote liver inflammation and metabolic dysfunction by causing liver pseudohypoxia (increased HIF-1α and HIF-2α expression in liver macrophages and hepatocytes). Increased HIF-1α and HIF-2α expression promotes pro-inflammatory activation of hepatic macrophages [107,127]. Furthermore, increased hepatocyte HIF-1α upregulates membrane-bound DPP4 expression and promotes sinusoidal vasoconstriction (which increases sinusoidal flow resistance), leading to increased first-pass inactivation of incretin hormones [89,128,129].

It is interesting to note that, while metaflammation propagates from adipocytes to infiltrating immune cells, depletion of adipocyte-specific TNF and NF-κB signaling at the early stages of obesity impairs insulin sensitivity and glucose tolerance [54,56,68], whereas inhibiting immune cell infiltration and pro-inflammatory activation (through adipocyte-specific depletion of HIF-1α or CCL2, or suppression of global or hemopoietic cell-specific TNF, JNK, and IKKβ/NF-κB signaling) improves insulin resistance and glucose intolerance in obese mice [15,63–65,83–88]. These findings raise the possibility that an acute inflammatory response within adipocytes at the early phase of obesity is metabolically beneficial (as far as it is contained in adipocytes); however, as obesity progresses, sustained immune activation and metaflammation become detrimental, causing adipose tissue dysfunction and systemic metabolic impairment.

In addition to local changes in adipose tissue and the liver, obesity also induces gut dysbiosis and increased intestinal permeability, facilitating the entry of microbiome-derived factors such as bacterial DNA, peptidoglycans, and lipopolysaccharides into the systemic circulation. These microbiome-derived factors promote innate immunity by stimulating Toll-like receptors (TLR4, TLR9) or NOD1 on the surface of macrophages, as well as adipocytes and hepatocytes, and promote metaflammation [57,130–132]. At the neuroendocrine level, central regulation of plasma glucocorticoid levels and autonomic signaling, together with local amplification of glucocorticoid action through increased 11beta-hydroxysteroid dehydrogenase type 1 expression in adipose tissue also contributes to sustaining an inflammatory milieu [133]. Moreover, obesity-associated disruption of circadian clock gene regulation also enhances aberrant pro-inflammatory cytokine production by immune cells [134].

In chronic obesity, these pro-inflammatory changes operate in concert with defective resolution programs. Resolution of inflammation is an active, coordinated process, rather than a passive dissipation of pro-inflammatory signals. It requires the clearance of dead cell debris (efferocytosis) and other inflammatory mediators (phagocytosis), the cessation of pro-inflammatory immune cell recruitment (such as monocytes and neutrophils), and the removal of these cells via lymphatic drainage. These events are orchestrated by a network of signaling molecules such as specialized pro-resolving mediators (SPMs), which are bioactive lipid compounds derived mainly from omega-3 fatty acids (e.g., DHA and EPA), and pro-resolving cytokines such as IL-10 [135,136]. Of interest, beyond terminating inflammation, SPMs can directly contribute to metabolic homeostasis by regulating adipocytes, hepatocytes, and skeletal myocyte function. In obesity, both SPM biosynthesis and signaling are impaired [137,138]. Accordingly, exogenous administration of select SPMs and IL-10 overexpression have both been shown to improve insulin resistance and metabolic profiles in obese mice [139–144].

Thus, the expansion and propagation of metaflammation are likely driven by multifactorial mechanisms. While each of the mechanisms can independently fuel metaflammation, they also reinforce one another, creating a feedforward loop in which the inhibition of one pathway can lead to broader improvements by alleviating others [6,116–119,122,145–158]. Over time, the cumulative burden of these changes drives progressive tissue damage and adipocyte death, reinforcing the self-perpetuating cycle for the expansion of metaflammation. It is important to note, however, that, as discussed earlier, not all facets of metaflammation necessarily lead to metabolic dysfunction. And the relative contribution of individual stressors and defective pro-resolving pathways can vary in determining the intensity, chronicity, and qualitative features of metaflammation. Therefore, each of the metaflammation-driving mechanisms should not be assumed to equally contribute to insulin resistance and metabolic dysfunction. This underscores the importance of delineating how each of these processes contribute to the adaptive versus maladaptive metaflammation and the transition between these states.

## Transition from ‘protective’ to ‘detrimental’ metaflammation

While molecular determinants for the transition from protective to detrimental metaflammation remain unclear, since the transition likely occurs as the severity and chronicity of obesity advances, it would be reasonable to assume that the accompanied pathologic changes may be responsible. As obesity progresses, adipose tissue shows a number of pathologic changes, including increased adipocyte death [159–164], adipose tissue fibrosis [62], cellular senescence [165], altered immune cell infiltration and phenotypic switching of the individual infiltrating immune cell types [166], vascular dysfunction [167], adipocyte iron accumulation [168], catecholamine resistance [169], alterations in adipose tissue-derived and circulating exosomes [170], and epigenetic reprogramming [171]. Subsequent adipose tissue dysfunction may also allow changes in the secretory profiles of adipose tissue and the exceeding of a critical lipotoxic threshold [3,172]. As these alterations are highly interconnected, disentangling causality, temporal hierarchy, and directionality among these processes often presents a classic causality dilemma and remains a challenge in the field. Nonetheless, phenotypic switching of immune cells, in particular macrophages, can explain many of these changes. The remainder of this Essay focuses on phenotypic switching of macrophages to obesity-specific phenotypes. This concept has been well-established in murine models through lineage tracing and genetic manipulation, although its translation to humans remains partial and is the focus of ongoing investigation.

### Phenotypic switching of ATMs during obesity

Among the various immune cell types involved in systemic and tissue-specific metabolic regulation that show functional shifts, macrophages represent the most abundant immune cells accumulating in adipose tissue, liver, skeletal muscle, and pancreatic islets in obesity [173–175]. Notably, macrophages are the primary cellular source of most pro-inflammatory cytokines and exosomes that regulate insulin sensitivity [18,176–178]. Systemic depletion of macrophages [49,179–181] or macrophage-specific manipulation of molecules within proximal inflammatory pathways (such as IKKβ, JNK, IRF5, CCR2, and HIF-1α) can improve insulin resistance and glucose intolerance in obese mice [25,65,126,182–184], suggesting that macrophages have a key role in the pathogenesis of insulin resistance in obesity. Recently, the importance of macrophages in driving adipose tissue inflammation and hepatic insulin resistance and steatosis was also shown using an interconnected microphysiological system containing adipocytes, hepatocytes, and macrophages derived from an isogenic human iPSC system [35].

In healthy states, the majority of ATMs are developmental yolk sac-derived resident ATMs [185]. Resident ATMs secrete factors that enhance insulin sensitivity, such as IL-10 and insulin-sensitizing exosomes [98,139,178] (Table 1). They also produce PDGFcc, which promotes adipocyte differentiation and lipid storage [186]. Moreover, resident ATMs are enriched in genes associated with endocytosis, lysosomal function, and cholesterol and iron metabolism, reflecting their critical role for sustaining adipose tissue homeostasis. For example, a subset of resident ATMs, characterized by high expression of the cholesterol transporter ABCA1 (*Abca*^*+*^ *Lyve1*^*+*^ *Tim4*^*+*^ ATMs) has a key role in post-prandial reverse cholesterol transport by facilitating high-density lipoprotein cholesterol efflux after a high-fat meal, thereby protecting against dyslipidemia and cardiovascular disease [187]. In addition, resident ATMs take up excessive extracellular iron and buffer local iron levels, preventing excessive iron accumulation in adipocytes, which causes insulin resistance [188,189].

**Table 1:** Metabolically beneficial vs. detrimental effects of distinct ATM subtypes in health and obesity.

	Resident ATMs	Recruited ATMs
**In health or the early stages of obesity (adaptive)**	Anti-inflammatory cytokine production; insulin-sensitizing exosome secretion; pro-angiogenic factor production (e.g., PDGF); NE clearance (SAMs); buffering local iron concentration (iron-handing ATMs); cholesterol reverse transport (ABCA1^+^ ATMs)	Pro-angiogenic ATMs (LYVE-1^+^ ATMs, *Ly6c*^+^ ATMs); clearing extracellular lipids and dead adipocyte debris (LAMs); supporting adaptive thermogenesis (ChAMs and *Cx3cr1*^+^ ATMs); resolution of inflammation (LAMs and ChAMs)
**In the late stages of obesity** **(maladaptive)**	Increased resident ATM death; pro-inflammatory activation; decreased IL-10 production; decreased secretion of insulin-sensitizing exosomes; decreased iron metabolism gene expression; increased NE clearance (SAMs); catecholamine resistance	Transition of LAMs to pro-inflammatory, M-IR ATMs; increased secretion of exosomes that induce insulin resistance and liver fibrosis

Abbreviations: ATM, adipose tissue macrophage; ChAM, acetylcholine-synthesizing ATM; LAM, lipid-associated ATM; M-IR, insulin resistance-inducing macrophage; NE, norepinephrine; SAM, sympathetic neuron-associated macrophage.

In obesity, ATMs undergo phenotypic switching from anti-inflammatory, insulin-sensitizing (M-IS) phenotypes to pro-inflammatory, insulin resistance-inducing (M-IR) phenotypes. Previously, it was suggested that the phenotypes of ATMs in health and obesity are similar to the M2- and M1-polarized bone marrow-derived macrophages, induced by either by IL-4/IL-13 (M2) or LPS/IFNγ (M1) [98] (Box 1). However, recent evidence indicates that ATMs do not exist in these narrow M1/M2 categories. Instead, they represent a highly heterogenous population, with each subtype displaying distinct transcriptomic signatures and functions. Accordingly, the M1/M2 classification system is no longer considered adequate to describe ATM phenotypes. Importantly, the obesity-induced phenotypic switching of ATMs is associated with functional changes in each ATM subtype, as well as with changes in the ratio between different ATMs subtypes. For example, resident ATMs retain some of their metabolically beneficial effects [187–190]; however, their relative abundance declines along with their insulin-sensitizing effects. This decline occurs concomitantly with the accumulation of recruited ATMs, comprising pro-inflammatory M-IR phenotypes, as well as pro-resolving phenotypes (discussed in detail in the following section). In obesity, chemokines and lipokines such as CCL2, CCL5, CCL8, S100, Sema3e, and leukotriene B4 promote monocyte recruitment into adipose tissue [82,97,98,191,192], where they differentiate into pro-inflammatory ATMs. In addition, obesity increases the expression of soluble factors that enhance retention of ATMs within adipose tissue, such as netrin and Sema3e, further sustaining ATM accumulation. Beyond promoting recruitment, CCL2 also stimulates local proliferation of ATMs. Thus, the expansion of the ATM pool arises from increased recruitment of circulating monocytes [98,193] and prolonged ATM retention [192,194,195], as well as enhanced local proliferation [99,196]. Genetic deletion or pharmacological inhibition of each of these ATM recruitment and retention mediators improves metaflammation, insulin sensitivity, and glucose tolerance in obese mice. Moreover, selective depletion of recruited macrophages improves metabolic profiles in obese mice [197], whereas the depletion of *Cd169*-expressing tissue-resident macrophages induces pathological adipose tissue remodeling and vascular dysfunction in lean mice, resulting in adipocyte hypertrophy and adipose tissue dysfunction [52]. Therefore, it would be reasonable to conclude that the decrease in the beneficial effects of M-IS ATMs, as well as the increase in M-IR ATMs is an etiologic component of insulin resistance and metabolic dysfunction in obesity.

**Box 1. M1/M2 macrophage polarization states and obesity-induced ATM activation.**

M1 macrophages, often referred to as ‘classically activated’ macrophages, represent a pro-inflammatory phenotype, characterized by the production of pro-inflammatory cytokines, reactive oxygen species, and nitric oxide [198]. By contrast, M2 macrophages, or ‘alternatively activated’ macrophages, display an anti-inflammatory phenotype that is primarily associated with tissue repair, remodeling, and resolution of inflammation [199,200]. Markers typically used to distinguish M1 and M2 macrophages, such as CD11c (M1) and CD206 (M2), are differentially expressed in healthy and obesity-induced adipose tissue macrophages (ATMs). Moreover, most resident ATMs express CD206, whereas recruited pro-inflammatory ATMs express CD11c. However, recent advances in single cell techniques have revealed that in vivo, ATMs do not exist in these narrow categories [29,48,53,201–205,206]. Additionally, in opposition to previous suggestions, CD11c and CD206 are not always reliable indicators for the pro- and anti-inflammatory states of ATMs or for recruited versus resident ATMs. For example, although CD206 is abundantly expressed in resident ATMs, it is also expressed in a subset of CD11c^+^ pro-inflammatory recruited ATMs. These CD206 and CD11c double-positive ATMs display highly pro-inflammatory phenotypes [207]. Furthermore, myeloid lineage-specific inhibition of IL-4 signaling, which induces M2 polarization in bone marrow-derived macrophages, suppresses adipose tissue inflammation with increased CD11c^+^ ATMs in mice [208]. In addition, CD206^+^ ATMs are increased in patients with diabetes and exhibit pro-inflammatory phenotypes in humans [209]. Furthermore, while CD11c^+^ ATMs represent pro-inflammatory, recruited ATMs in the context of obesity, during weight loss, CD11c^+^ ATMs exhibit substantially reduced pro-inflammatory gene expression without losing CD11c expression [72]. Therefore, despite some similarities, M2 is not representative of healthy insulin-sensitizing ATMs nor is M1 representative of insulin resistance-inducing ATMs. Instead, ATMs in obesity exhibit unique metabolic gene expression profiles characterized by elevated expression of genes involved in lipid metabolism, as well as mitochondrial and lysosomal activities, which is termed the ‘metabolic activation state (MMe)’ [202–204].

### Specific changes in distinct ATM subtypes

#### Secretion of insulin-sensitizing versus insulin resistance-inducing exosomes.

Exosomes are nano-sized extracellular vesicles that encapsulate diverse bioactive intracellular cargo, including lipids, proteins, mRNAs, microRNAs, long noncoding RNAs, and functional mitochondrial fragments. They are released into extracellular space, where they can subsequently enter systemic circulation [210]. By transferring these intracellular contents from the originating cells, exosomes mediate intercellular communication and modulate recipient cell function [210–214]. Adipose tissue represents a major source of circulating exosomes [170,215]. Treatment with plasma or adipose tissue-derived exosomes from obese individuals with MASLD, but not from lean or obese individuals without MASLD, impairs insulin signaling in cultured human skeletal muscle myotubes and primary mouse hepatocytes [37]. This effect correlates with skeletal muscle and hepatic insulin sensitivity of the donors [69]. While adipocytes are thought to be the major contributors of adipose tissue-derived exosomes [215], ATMs also secrete exosomes that regulate insulin sensitivity in adipocytes, hepatocytes, and skeletal myotubes [178,216,217]. Notably, in healthy mice maintained on a normal chow diet, ATM-derived small extracellular vesicles containing exosomes enhance insulin sensitivity, an effect observed in ATMs from both VAT and SAT. By contrast, in obese mice, VAT ATMs secrete exosomes that promote insulin resistance [178], whereas SAT ATMs largely retain their capacity to secrete exosomes that improve insulin resistance. This VAT-specific phenotypic switching of ATM-derived exosomes is associated with a decrease in resident ATMs, which retain their insulin-sensitizing effect in obesity, as well as the increase in recruited M-IR ATMs. These changes are mediated, at least in part, by CCL26 derived from subcutaneous adipocytes and adipocyte progenitors and by CXCL12 secreted from SAT-resident ATMs [79]. CCL26 increases M-IS and decreases M-IR ATMs by decreasing resident ATM death and blood monocyte chemotaxis. CXCL12 educates recruited ATMs to secret insulin-sensitizing small extracellular vesicles. Notably, treatment with the insulin-sensitizing agent, rosiglitazone, or with CXCL12 reverses this switch, restoring the beneficial effects of ATM-derived exosomes [79,178,190,217]. Although exosomes carry various classes of bioactive molecules, inhibition of microRNA biogenesis in ATMs via Dicer depletion blunts the effect of ATM-derived exosomes on insulin sensitivity, suggesting that microRNAs are the key mediators of this effect. Several microRNAs contained within ATM-derived exosomes have been identified as regulators of insulin sensitivity, including miR-690, miR-155, and miR-210 [178,216–218].

#### Iron-handling ATMs.

Iron is essential for mitochondrial function and cellular bioenergetics. However, excess iron promotes oxidative stress through metal-catalyzed reaction, in particular the Fenton reaction, which generates ROS. This oxidative burden can drive mitochondrial dysfunction and contribute to damage in vital organs such as the liver, heart, and pancreas. Insulin limits post-prandial increases in circulating iron levels by suppressing intestinal iron absorption and reducing iron export from hepatocytes, macrophages, and adipocytes through upregulating hepcidin expression, which in turn inhibits ferroportin activity [219]. Iron overload is frequently observed in patients with T2D or impaired glucose tolerance. In obesity, adipose tissue iron levels increase. In mice with adipocyte-specific depletion of ferroportin, increased adipocyte iron accumulation worsens insulin resistance and glucose intolerance in obesity [220]. Conversely, reducing adipocyte iron level by adipocyte-specific depletion of transferrin receptor protects from the development of insulin resistance and glucose intolerance with decreased weight gain in mice fed a HFD [221], suggesting that adipocyte iron overload leads to adipose tissue dysfunction and insulin resistance. Of note, adipocyte iron levels are critically regulated by a subset of resident ATMs, termed MFe^hi^ ATMs. MFe^hi^ ATMs exhibit high expression of iron storage and metabolism genes and prevent adipocyte iron overload by sequestering excess extracellular iron [188,189]. In obesity, the number of MFe^hi^ ATMs is reduced along with decreased expression of genes involved in iron metabolism, facilitating iron accumulation in adipocytes [188,222,223].

Of interest, although ATM labile iron content is decreased, total intracellular iron content is generally increased in ATMs along with increased oxidative stress [222]. Genetic manipulation that increases iron content in the mitochondrial matrix of macrophages exacerbates pro-inflammatory ATM activation, adipocyte iron accumulation, insulin resistance, and glucose intolerance in obese mice [222]. Conversely, reducing macrophage mitochondrial matrix iron content improves insulin sensitivity and glucose tolerance together with attenuated metaflammation, increased anti-inflammatory ATM activation, and decreased adipocyte iron accumulation. Therefore, it is likely that aberrant iron accumulation in ATMs also causes pro-inflammatory ATM activation and insulin resistance in obesity. As a possible explanation for the obesity-induced decrease in MFe^hi^ resident ATMs and iron-induced pro-inflammatory ATM activation, we recently observed that obesity induces ferroptosis in resident ATMs [190], which may contribute to the relative depletion of MFe^hi^ iron-handling ATM subset and the replacement of them with pro-inflammatory recruited ATMs.

#### ATMs that clear dead adipocyte debris and extracellular lipids.

In healthy adult humans, adipocytes exhibit a slow turnover rate with ~10% of the adipocyte pool renewed annually through apoptosis [161,224]. Macrophages are among the best-known professional phagocytes. Like other tissue macrophages, ATMs engulf and degrade extracellular debris (such as dead adipocytes; i.e., efferocytosis) and foreign particles [161,204]. Resident ATMs in lean adipose tissue are characterized by high expression of genes involved in lysosomal activity and phagocytosis. They aggregate around dead adipocytes, forming histologically distinctive crown-like structures for effective efferocytosis. In obesity, however, the rate of (pre)adipocyte death increases through a combination of programmed and nonprogrammed cell death mechanisms, including pyroptosis, necrosis, apoptosis, and ferroptosis [159–164]. Dying adipocytes release damage-associated molecular patterns, such as ATP, uric acid, and nucleic acids, which are recognized by pattern recognition receptors such as TLRs and NOD-like receptors on macrophages and other immune cells. This recognition triggers local inflammatory responses that alter the secretory profile of white adipocytes (adipocytokines) and impair adaptive thermogenesis in brown/beige adipocytes [225]. Furthermore, lipids released in an uncontrolled manner from dead adipocytes can enter the systemic circulation and be deposited ectopically in nonadipose organs such as the liver, skeletal muscle, and heart, inducing mitochondrial stress, ER stress, insulin resistance, and broader metabolic dysfunction (lipotoxicity). However, in obesity, the abundance and phagocytic activities of resident ATMs decline due to HIF-2α-dependent activation of the mTOR pathway, which suppresses lysosome gene expression, thereby impairing phago-/efferocytic activities while enhancing lysosomal cell death [107]. Instead, a distinct subset of recruited ATMs specialized in clearing extracellular lipids derived from increased lipolysis or dead adipocytes emerges. These ATMs express high levels of genes involved in lipid metabolism (such as *Pparg, p62, Trem2, Cd9,* and *Cd36*), mitochondrial oxidative phosphorylation, and lysosomal activity (such as *Lamp2*, *Acp5*, and *Ctsk*), equipping them for efficient lipid uptake, degradation, and clearance. These ATMs take up fatty acids through caveolae-dependent endocytosis mediated by scavenger receptors, such as CD36. They also perform exophagy, a process by which lysosomes are released via exocytosis to degrade apoptotic adipocytes [226].

Several independent studies have described these obesity-induced, circulating monocyte-derived ATMs with largely overlapping phenotypes under different names, including MMe ATMs (Box 1) and lipid-associated ATMs (LAMs) [29,53,202,203]. scRNA-seq data analyses indicate that these populations are largely identical (or, at a minimum, extensively overlapping). Despite variations in the specific markers or criteria used across studies, for convenience and consistency, we hereafter refer to this population as LAMs. LAM phenotypes are likely shaped by excess fatty acids (notably, palmitic acid) and adipocyte-derived factors, such as lipid-laden small extracellular vesicles [202,227]. In obesity, LAMs accumulate, particularly around crown-like structures (marking sites of dead adipocytes) [53]. Moreover, increased lipolysis during weight loss leads to transient accumulation of LAMs in adipose tissue [71]. Functional studies using mice with global or myeloid lineage-specific depletion of LAM-specific regulators/markers, such as Trem2 [53], TFEB [228], or PPARγ [229], suggest that LAMs protect against the development of insulin resistance and glucose tolerance in obesity. For example, global Trem2-deficient mice develop adipocyte hypertrophy, increased body fat accumulation, and glucose intolerance [53,230], although other studies failed to confirm metabolic beneficial effects of global or macrophage-specific Trem2 [231,232].

Although increased in obesity, the effect of LAMs on clearing dead adipocytes and extracellular lipids is likely not sufficient to rescue from metaflammation, insulin resistance, and metabolic dysfunction. Thus, obesity increases the expression of the lysosomal protein TM4SF19 in ATMs, which represses acidification through its interaction with vacuolar ATPase. TM4SF19 inactivation elevates lysosomal acidification and increases the clearance of dead adipocytes. Mice lacking TM4SF19 show improved insulin sensitivity in obesity [233]. Similarly, boosting ATM lysosomal activity by myeloid lineage-specific overexpression of the lysosomal master transcription factor TFEB protects against adipose tissue inflammation and insulin resistance in obese mice, whereas myeloid lineage-specific TFEB depletion worsens adipose tissue inflammation and insulin resistance [228]. Furthermore, suppressing mTOR-dependent inhibition of TFEB via antisense oligonucleotides targeting Folliculin ameliorates metaflammation and metabolic dysfunction-associated steatohepatitis (MASH) in obese mice [107]. Therefore, although it may not be sufficient to fully prevent metaflammation and adipose tissue dysfunction, increased lysosomal activity in LAMs likely helps suppress them by clearing dead adipocytes and excess extracellular lipids. Since autophagy is increased in ATMs in obesity [234], blocks pro-inflammatory ATM activation, and protects against insulin resistance and glucose intolerance in obese mice [235], lysosomal activity in ATMs in obesity may help limit metaflammation by enhancing autophagy and inhibiting pro-inflammatory ATM activation.

It is interesting to note that, while LAMs are generally considered to be a pro-resolving, protective ATM subtype, they are not quiescent ATMs. Thus, LAMs express higher levels of pro-inflammatory genes compared with resident ATMs [53,79,204], although they exhibit relatively lower expression of pro- inflammatory genes compared with other *Cd9*-expressing, recruited ATMs [53,230–232]. Indeed, LAMs show substantial overlap with previously defined CD11c^+^ pro-inflammatory, M-IR ATMs in visceral white adipose tissue from obese mice [53,79,125]. One study in mice identified NOX2 as a key driver of both pro- inflammatory and adipocyte-clearing properties of LAMs and showed that depletion of NOX2 attenuates pro-inflammatory ATM activation and improves glucose tolerance after 8 weeks of a HFD while, paradoxically, it worsens insulin resistance with the accumulation of dead adipocytes after 16 weeks of a HFD [204]. These results suggest that although LAMs are generally considered to be a pro-resolving, protective ATM subtype, they may also mediate metabolically detrimental effects during the development of obesity. Interestingly, a recent study showed that LAMs can transition into pro-inflammatory ATMs during the progression of obesity [79], mirroring their phenotypic shift in atherosclerosis [236]. Therefore, it is possible that LAMs may represent an intermediate state, in which persistent metabolic stress (or failure to adequately control extracellular lipid and dead adipocyte accumulation) drives their conversion into pro-inflammatory ATMs. To date, most funtional studies have investigated the effects of LAM-specific molecules (e.g., *Trem2*), instead of the role of LAMs themselves. Additionally, *Trem2* and *Nox2* are not exclusively expressed in LAMs, but are also expressed in other macrophage subtypes, such as resident ATMs, although they are more abundant in LAMs. Therefore, future studies are required to define the specific effects of this ATM subpopulation on the development of metaflammation and insulin resistance, and to understand how the protective effects of LAMs decline to favor pro-inflammatory phenotypes.

#### ATMs that metabolize catecholamine and acetylcholine and regulate lipolysis and adaptive thermogenesis.

One of the primary functions of adipose tissue is to store excess lipids during periods of caloric surplus and to release them in accordance with metabolic demands. For example, during fasting or cold exposure, activation of the sympathetic nervous system (SNS) in adipose tissue triggers the release of norepinephrine (NE) from local sympathetic nerves. Acting through beta adrenergic receptors on adipocytes, NE promotes lipolysis in white adipocytes and drives adaptive thermogenesis in brown and beige adipocytes. In addition, NE inhibits adipose tissue expansion by suppressing the proliferation and differentiation (adipogenesis) of preadipocytes, while it enhances sympathetic innervation by stimulating the recruitment of eosinophils, which secrete nerve growth factor [237]. A specialized subset of resident ATMs, known as sympathetic neuron-associated macrophages (SAMs), resides in close proximity to sympathetic nerves within adipose tissue. These ATMs take up and catabolize catecholamines (including NE) [238], which may help limit excessive energy expenditure and enable rapid recovery of the capacity to expand adipose tissue. However, in the context of obesity and aging, an increased number of or activity of SAMs leads to reduced adaptive thermogenic capacity [238,239].

In addition to SAMs, a *Cx3cr1*-expressing ATM subtype that resides in brown adipose tissue has an essential role in sympathetic innervation into interscapular brown adipose tissue in mice [240]. In contrast to the function of SAMs, it has also been suggested that anti-inflammatory M2-like polarized ATMs can directly generate catecholamines, and that treatment with IL-4 promotes adaptive thermogenesis by inducing M2-like polarization of ATMs [241,242]. However, this latter hypothesis has been challenged by subsequent studies [243].

Similar to catecholamines, acetylcholine (ACh), the primary neurotransmitter of the parasympathetic nervous system, also enhances thermogenesis. Of note, adipose tissue lacks parasympathetic innervation [244]. Instead, adipose tissue ACh is produced from a distinct subset of ATMs, called acetylcholine-synthesizing ATMs (ChAMs). ChAMs constitute a minor fraction (~0.17%) of stromal vascular cells in inguinal adipose tissue [245,246]. They release ACh in response to NE stimulation and enhance the cAMP–PKA pathway and adaptive thermogenesis in beige adipocytes in subcutaneous adipose tissue [245,246]. Myeloid lineage-specific genetic deletion of choline acetyltransferase (*Chat*), the rate-limiting enzyme for acetylcholine synthesis, impairs cold-induced adaptive thermogenesis and reduces cold tolerance in lean, healthy mice. Although it is not known whether obesity affects the abundance or functional activity of ChAMs, depletion of cholinergic receptor nicotinic alpha 2 subunit (*Chrna2*) in *Ucp1*-expressing brown/beige adipocytes exacerbates diet-induced obesity in mice fed a HFD. Therefore, local cholinergic signaling in adipose tissue (likely mediated by ChAMs) may exerts an anti-obesogenic role under conditions of nutrient excess.

In support of the concept that ATMs regulate local catecholamine and ACh availability, systemic depletion of tissue macrophages using clodronate liposomes, but not sympathetic denervation, abolished beiging of subcutaneous adipose tissue in adipocyte-specific *Fasn* knockout mice [247]. Of note, although both NE and ACh promote thermogenic responses in brown/beige adipocytes and ChAMs are activated by NE, ACh suppresses TNF-induced pro-inflammatory gene expression while increasing *Glut4* expression in adipocytes [248], whereas NE promotes acute inflammation by increasing lipolysis and FFA levels [249]. Moreover, ACh enhances efferocytosis by macrophages and the resolution of inflammation [250]. Therefore, it is likely that distinct subsets of ATMs actively regulate lipolysis, adaptive thermogenesis, and adipose tissue mass by regulating sympathetic innervation and local NE and ACh levels.

It remains unclear how these macrophage-mediated regulation of local NE and ACh levels and their downstream effects are coordinated in obesity. Central regulation of sympathetic innervation of adipose tissue is decreased in obesity due to leptin resistance in the paraventricular nucleus of the hypothalamus, which can be reversed by chronic leptin treatment [251]. Given that obesity also reduces adipose eosinophil infiltration while increasing SAMs, these local immune changes may further decrease sympathetic innervation, lower local NE levels, and suppress ChAM activation, which can collectively enhance catecholamine resistance and impair adaptive thermogenesis.

#### ATMs that regulate angiogenesis and tissue remodeling during adipose tissue expansion.

In order to store excess lipids and release them according to metabolic demand, mature adipocytes can adjust their volume by up to 100-fold [224,252,253]. In addition, both adult humans and mice retain the capacity for de novo adipogenesis throughout life, which is increased in obesity. To maintain adequate perfusion and efficient delivery of oxygen and nutrients, adipose tissue expansion is coordinated with angiogenesis and vascular remodeling [62]. Macrophages secrete VEGF, PDGF, matrix metalloproteinases, TNF, and other cytokines. These factors facilitate not only endothelial cell migration, proliferation, and new blood vessel formation, but also degradation of extracellular matrix to allow vessel sprouting and remodeling [254,255]. Resident ATMs abundantly express these factors and the depletion of tissue-resident macrophages causes vascular dysfunction [52]. In obesity, the resident ATM population declines, whereas a subset of ATMs expressing LYVE-1 accumulates preferentially around the dense vascular network located at the leading edge of expanding adipose tissue [256–258]. These LYVE-1^+^ ATMs serve as an important source of PDGF, necessary for angiogenic remodeling. The depletion of macrophages using clodronate liposomes reduces the formation of a dense vascular network, underscoring the role of ATMs in adipose tissue expansion. LYVE-1^+^ ATMs are recruited from bone marrow (i.e., recruited ATMs) by local hypoxia-induced CXCL12 expression, independent of the CCR2–CCL2 system [256]. Similar to these LYVE-1^+^ ATMs, scRNA-seq analyses revealed that a subset of recruited ATMs expressing a monocyte marker, *Lyc6*, most abundantly express pro-angiogenic genes compared with other ATM subtypes including Trem2^+^ and/or CD9^+^ populations [29]. These ATMs are located outside of the crown-like structure and also enhance adipocyte differentiation, consistent with their role in adipose tissue expansion. A subset of ATMs displaying pro-angiogenic gene signatures was also identified in humans [259].

While the longitudinal dynamics of pro-angiogenic ATM abundance and function across the full course of obesity remain incompletely defined, it is noteworthy that the expression of pro-angiogenic factors and their activity within adipose tissue acutely increase at an early stage during the development of obesity [84], but eventually decline together with decreased vascular density and function within adipose tissue in chronic obesity [92,93], contributing to adipose tissue hypoxia. Adipocyte-specific overexpression of VEGF-A or VEGF-B improves insulin sensitivity and glucose tolerance with increased vascular density and oxygen tension in adipose tissue in obese mice. Conversely, adipocyte-specific depletion of VEGF-A worsens adipose tissue inflammation, insulin resistance and glucose intolerance [93–96]. Interestingly, although pro-angiogenic stimulation supports healthy adipose tissue remodeling at the early stages of obesity, inhibition of VEGF signaling or angiogenesis after the onset of obesity can also yield metabolic benefits, including reducing adiposity and body weight and improved adipose tissue inflammation and glucose tolerance [93,260–264]. Therefore, it is likely that the metabolic consequences of pro-angiogenic signaling in adipose tissue are context- and timing-dependent. It still remains to be elucidated whether and how pro-angiogenic ATM abundance and activity eventually decline during chronic obesity and the molecular determinants distinguishing the beneficial versus detrimental effects of angiogenic processes on metabolic health.

## Conclusions and future directions

In summary, although metaflammation ultimately leads to insulin resistance and metabolic dysfunction in chronic obesity, it likely begins as an adaptive response to increased oxygen demand and metabolic stress rather than as a purely pathological process. During the early stages of obesity, metaflammation does not cause insulin resistance. Instead, metaflammation, with the activation of distinct resident ATM subtypes (including pro-angiogenic ATMs and iron-handling ATMs), appears to support healthy adipose tissue expansion and to restrain the progression of insulin resistance and metabolic dysfunction. In addition, SAMs may contribute to clearing extracellular NE, thereby limiting excessive lipolysis and sustaining adipose tissue expendability. At this stage, recruited ATMs that differentiate into ChAMs and LAMs also promote metabolic homeostasis by enhancing energy expenditure and buffering extracellular lipid accumulation. As obesity progresses, however, the beneficial effects of resident ATMs in secreting insulin-sensitizing exosomes, anti-inflammatory cytokines (such as IL-10), and pro-angiogenic and adipogenic factors (e.g., PDGF) gradually decline, as does the iron-handling capacity of resident ATMs. At the same time, sustained resident SAM activation may suppress adaptive thermogenesis and promote weigh gain, and recruited ATMs such as LAMs can transition into pro-inflammatory M-IR ATMs, which secrete cytokines and exosomes that directly induce insulin resistance. Collectively, these changes likely contribute to the shift of metaflammation from an adaptive process to a chronic, maladaptive, nonresolving state, driving insulin resistance, and metabolic dysfunction. Thus, just as adipose tissue itself is not inherently pathological but is critical for metabolic homeostasis, not all macrophage-mediated or inflammatory responses should be viewed as detrimental. Rather, the failure of beneficial functions of metaflammation to maintain metabolic homeostasis and the pathological reprogramming of ATMs into M-IR phenotypes may represent central etiologic contributors to the development of insulin resistance and metabolic dysfunction in obesity.

Genetic and pharmacologic studies targeting proximal inflammatory pathways have established chronic tissue metaflammation as a key contributor to the pathogenesis of insulin resistance and glucose intolerance. However, most of these studies were conducted in mice. In humans, obesity is associated with increased infiltration of pro-inflammatory immune cells, including macrophages, in adipose tissue, liver, skeletal muscle, and pancreatic islets, and in vitro work in primary human adipocytes, hepatocytes, skeletal myotubes, and organoids is consistent with a causal role for metaflammation. However, because the types of genetic manipulation used in murine models are not feasible in humans, pharmacologic interventions are required to rigorously test whether modifying metaflammation can prevent or reverse metabolic dysfunction.

To date, several anti-inflammatory strategies directed against targets such as TNF, CCL2, and IL-1β have been tested for the treatment of insulin resistance and T2D. Although some have shown positive results, most yielded disappointing efficacy and raised safety concerns [19,48]. While some clinically available insulin-sensitizing agents, including thiazolidinediones and salicylic acid derivatives, can directly suppress inflammatory pathways and improve markers of metaflammation in parallel with better metabolic profiles, it remains unclear to what extent their metabolic benefits are mediated by direct immunomodulation versus other mechanisms. Because not all inflammatory responses during metaflammation are deleterious and acute activation of selected pathways can support healthy adipose tissue expansion and promote resolution of chronic inflammation, future therapeutic strategies should aim to distinguish and selectively modulate adaptive versus pathogenic inflammatory programs. Ideally, interventions would target the molecular determinants governing the transition between these states while minimizing systemic safety concerns. In this context, drug-delivery platforms capable of selectively targeting adipocytes or defined macrophage subsets represent a particularly attractive approach to enhance efficacy and limit off-target (or unwanted on-target) effects.

As obesity is a major risk factor for metaflammation, insulin resistance, and downstream complications including T2D, MASH, and related metabolic disorders, sustained weight loss would be a rational therapeutic goal. However, durable weight reduction through lifestyle interventions alone is difficult to achieve in most cases. Pharmacological interventions such as GLP-1/GIP-based pharmacotherapies provide an effective option for weight reduction and improving metabolic parameters [265]. However, their broader utility can be limited by adverse effects (including gastrointestinal side effects), cost, and access, and the frequent occurrence of rapid weight regain upon discontinuation. Weight regain is often followed by prompt recurrence of metaflammation, insulin resistance, and metabolic dysfunction, suggesting that immunologic and tissue level memory of obesity and metaflammation may persist despite transient weight loss [266]. A detailed mechanistic understanding of how chronic metaflammation remodels metabolic tissues will also be essential to identify durable points of intervention.

Overall, understanding how metaflammation is initiated, expands, propagates, and is ultimately converted into a driver of metabolic disease remains a challenge for the field. Key unresolved questions include: how pro-inflammatory factors and lipid mediators differ in beneficial versus detrimental phases of metaflammation; which novel pathological pathways and cellular players emerge during the transition; by what mechanisms protective inflammation transitions to maladaptive responses; how resolution of inflammation fails and pro-inflammatory signaling persists; and whether these processes are reversible or instead embedded through epigenetic, microenvironmental, or immune memory-related mechanisms. As these mechanisms are elucidated, hopefully, there will be an opportunity to develop more precise tools that can test the causal hypothesis in humans.

## Abbreviations

ACh: acetylcholine
ATMs: adipose tissue macrophages
ChAM: acetylcholine-synthesizing ATM
ER: endoplasmic reticulum
FFA: free fatty acid
HFD: high-fat diet
IKKβ: IκB kinase β
LAMs: lipid-associated ATMs
MASLD: metabolic dysfunction-associated steatotic liver disease
MASH: metabolic dysfunction-associated steatohepatitis
MMe: metabolic activation state
M-IR: insulin resistance-inducing macrophage
NE: norepinephrine
NK: natural killer cells
NO: nitric oxide
ROS: reactive oxygen species
SAMs: sympathetic neuron-associated macrophages
SAT: subcutaneous adipose tissue
SNS: sympathetic nervous system
SPMs: specialized pro-resolving mediators
T2D: type 2 diabetes mellitus
Treg: regulatory T

## References

[1] NCD Risk Factor Collaboration (NCD-RisC). Worldwide trends in underweight and obesity from 1990 to 2022: a pooled analysis of 3663 population-representative studies with 222 million children, adolescents, and adults. Lancet. 2024;403(10431):1027–50. doi: 10.1016/S0140-6736(23)02750-2 38432237 PMC7615769

[2] Klein S, Gastaldelli A, Yki-Järvinen H, Scherer PE. Why does obesity cause diabetes? Cell Metab. 2022;34(1):11–20. doi: 10.1016/j.cmet.2021.12.012 34986330 PMC8740746

[3] Roden M, Shulman GI. The integrative biology of type 2 diabetes. Nature. 2019;576(7785):51–60. doi: 10.1038/s41586-019-1797-8 31802013

[4] Sangwung P, Petersen KF, Shulman GI, Knowles JW. Mitochondrial dysfunction, insulin resistance, and potential genetic implications. Endocrinology. 2020;161(4):bqaa017. doi: 10.1210/endocr/bqaa017 32060542 PMC7341556

[5] Newgard CB. Metabolomics and metabolic diseases: where do we stand? Cell Metab. 2017;25(1):43–56. doi: 10.1016/j.cmet.2016.09.018 28094011 PMC5245686

[6] Halban PA, Polonsky KS, Bowden DW, Hawkins MA, Ling C, Mather KJ, et al. β-cell failure in type 2 diabetes: postulated mechanisms and prospects for prevention and treatment. Diabetes Care. 2014;37(6):1751–8. doi: 10.2337/dc14-0396 24812433 PMC4179518

[7] Czech MP. Insulin action and resistance in obesity and type 2 diabetes. Nat Med. 2017;23(7):804–14. doi: 10.1038/nm.4350 28697184 PMC6048953

[8] Guilherme A, Henriques F, Bedard AH, Czech MP. Molecular pathways linking adipose innervation to insulin action in obesity and diabetes mellitus. Nat Rev Endocrinol. 2019;15(4):207–25. doi: 10.1038/s41574-019-0165-y 30733616 PMC7073451

[9] Alonge KM, D’Alessio DA, Schwartz MW. Brain control of blood glucose levels: implications for the pathogenesis of type 2 diabetes. Diabetologia. 2021;64(1):5–14. doi: 10.1007/s00125-020-05293-3 33043401 PMC7718404

[10] Scherer PE. The many secret lives of adipocytes: implications for diabetes. Diabetologia. 2019;62(2):223–32. doi: 10.1007/s00125-018-4777-x 30465066 PMC6324990

[11] DeFronzo RA, Ferrannini E, Groop L, Henry RR, Herman WH, Holst JJ, et al. Type 2 diabetes mellitus. Nat Rev Dis Primers. 2015;1:15019. doi: 10.1038/nrdp.2015.19 27189025

[12] Kahn CR, Wang G, Lee KY. Altered adipose tissue and adipocyte function in the pathogenesis of metabolic syndrome. J Clin Invest. 2019;129(10):3990–4000. doi: 10.1172/JCI129187 31573548 PMC6763230

[13] Hotamisligil GS. Inflammation, metaflammation and immunometabolic disorders. Nature. 2017;542(7640):177–85. doi: 10.1038/nature21363 28179656

[14] Feingold KR, Serio MK, Adi S, Moser AH, Grunfeld C. Tumor necrosis factor stimulates hepatic lipid synthesis and secretion. Endocrinology. 1989;124(5):2336–42. doi: 10.1210/endo-124-5-2336 2707158

[15] Hotamisligil GS, Shargill NS, Spiegelman BM. Adipose expression of tumor necrosis factor-alpha: direct role in obesity-linked insulin resistance. Science. 1993;259(5091):87–91. doi: 10.1126/science.7678183 7678183

[16] Sethi JK, Hotamisligil GS. Metabolic messengers: tumour necrosis factor. Nat Metab. 2021;3(10):1302–12. doi: 10.1038/s42255-021-00470-z 34650277

[17] Xu H, Barnes GT, Yang Q, Tan G, Yang D, Chou CJ, et al. Chronic inflammation in fat plays a crucial role in the development of obesity-related insulin resistance. J Clin Invest. 2003;112(12):1821–30. doi: 10.1172/JCI19451 14679177 PMC296998

[18] Weisberg SP, McCann D, Desai M, Rosenbaum M, Leibel RL, Ferrante AW Jr. Obesity is associated with macrophage accumulation in adipose tissue. J Clin Invest. 2003;112(12):1796–808. doi: 10.1172/JCI19246 14679176 PMC296995

[19] Goldfine AB, Shoelson SE. Therapeutic approaches targeting inflammation for diabetes and associated cardiovascular risk. J Clin Invest. 2017;127(1):83–93. doi: 10.1172/JCI88884 28045401 PMC5199685

[20] Donath MY, Dinarello CA, Mandrup-Poulsen T. Targeting innate immune mediators in type 1 and type 2 diabetes. Nat Rev Immunol. 2019;19(12):734–46. doi: 10.1038/s41577-019-0213-9 31501536

[21] Zhao P, Wong KI, Sun X, Reilly SM, Uhm M, Liao Z, et al. TBK1 at the crossroads of inflammation and energy homeostasis in adipose tissue. Cell. 2018;172(4):731-743.e12. doi: 10.1016/j.cell.2018.01.007 29425491 PMC5808582

[22] Hall JA, Ramachandran D, Roh HC, DiSpirito JR, Belchior T, Zushin P-JH, et al. Obesity-linked PPARγ S273 phosphorylation promotes insulin resistance through growth differentiation factor 3. Cell Metab. 2020;32(4):665-675.e6. doi: 10.1016/j.cmet.2020.08.016 32941798 PMC7543662

[23] Xia JY, Holland WL, Kusminski CM, Sun K, Sharma AX, Pearson MJ, et al. Targeted induction of ceramide degradation leads to improved systemic metabolism and reduced hepatic steatosis. Cell Metab. 2015;22(2):266–78. doi: 10.1016/j.cmet.2015.06.007 26190650 PMC4527941

[24] Arkan MC, Hevener AL, Greten FR, Maeda S, Li Z-W, Long JM, et al. IKK-beta links inflammation to obesity-induced insulin resistance. Nat Med. 2005;11(2):191–8. doi: 10.1038/nm1185 15685170

[25] Weisberg SP, Hunter D, Huber R, Lemieux J, Slaymaker S, Vaddi K, et al. CCR2 modulates inflammatory and metabolic effects of high-fat feeding. J Clin Invest. 2006;116(1):115–24. doi: 10.1172/JCI24335 16341265 PMC1307559

[26] Hirosumi J, Tuncman G, Chang L, Görgün CZ, Uysal KT, Maeda K, et al. A central role for JNK in obesity and insulin resistance. Nature. 2002;420(6913):333–6. doi: 10.1038/nature01137 12447443

[27] Fabbrini E, Cella M, McCartney SA, Fuchs A, Abumrad NA, Pietka TA, et al. Association between specific adipose tissue CD4+ T-cell populations and insulin resistance in obese individuals. Gastroenterology. 2013;145(2):366-74.e1-3. doi: 10.1053/j.gastro.2013.04.010 23597726 PMC3756481

[28] Löfgren P, van Harmelen V, Reynisdottir S, Näslund E, Rydén M, Rössner S, et al. Secretion of tumor necrosis factor-alpha shows a strong relationship to insulin-stimulated glucose transport in human adipose tissue. Diabetes. 2000;49(5):688–92. doi: 10.2337/diabetes.49.5.688 10905474

[29] Hill DA, Lim H-W, Kim YH, Ho WY, Foong YH, Nelson VL, et al. Distinct macrophage populations direct inflammatory versus physiological changes in adipose tissue. Proc Natl Acad Sci U S A. 2018;115(22):E5096–105. doi: 10.1073/pnas.1802611115 29760084 PMC5984532

[30] Senn JJ, Klover PJ, Nowak IA, Mooney RA. Interleukin-6 induces cellular insulin resistance in hepatocytes. Diabetes. 2002;51(12):3391–9. doi: 10.2337/diabetes.51.12.3391 12453891

[31] Ghazarian M, Revelo XS, Nøhr MK, Luck H, Zeng K, Lei H, et al. Type I interferon responses drive intrahepatic T cells to promote metabolic syndrome. Sci Immunol. 2017;2(10):eaai7616. doi: 10.1126/sciimmunol.aai7616 28567448 PMC5447456

[32] Liu LS, Spelleken M, Röhrig K, Hauner H, Eckel J. Tumor necrosis factor-alpha acutely inhibits insulin signaling in human adipocytes: implication of the p80 tumor necrosis factor receptor. Diabetes. 1998;47(4):515–22. doi: 10.2337/diabetes.47.4.515 9568681

[33] Austin RL, Rune A, Bouzakri K, Zierath JR, Krook A. siRNA-mediated reduction of inhibitor of nuclear factor-κB kinase prevents tumor necrosis factor-α-induced insulin resistance in human skeletal muscle. Diabetes. 2008;57(8):2066–73. doi: 10.2337/db07-0763 18443205 PMC2494681

[34] Maedler K, Sergeev P, Ris F, Oberholzer J, Joller-Jemelka HI, Spinas GA, et al. Glucose-induced beta cell production of IL-1beta contributes to glucotoxicity in human pancreatic islets. J Clin Invest. 2002;110(6):851–60. doi: 10.1172/JCI15318 12235117 PMC151125

[35] Qi L, Groeger M, Sharma A, Goswami I, Chen E, Zhong F, et al. Adipocyte inflammation is the primary driver of hepatic insulin resistance in a human iPSC-based microphysiological system. Nat Commun. 2024;15(1):7991. doi: 10.1038/s41467-024-52258-w 39266553 PMC11393072

[36] Pickup JC, Mattock MB, Chusney GD, Burt D. NIDDM as a disease of the innate immune system: association of acute-phase reactants and interleukin-6 with metabolic syndrome X. Diabetologia. 1997;40(11):1286–92. doi: 10.1007/s001250050822 9389420

[37] Samovski D, Smith GI, Palacios H, Pietka T, Fuchs A, Patti GJ, et al. Effect of marked weight loss on adipose tissue biology in people with obesity and type 2 diabetes. Diabetes Care. 2025;48(8):1342–51. doi: 10.2337/dc24-2739 40208704 PMC12281976

[38] Petersen MC, Smith GI, Palacios HH, Farabi SS, Yoshino M, Yoshino J, et al. Cardiometabolic characteristics of people with metabolically healthy and unhealthy obesity. Cell Metab. 2024;36(4):745-761.e5. doi: 10.1016/j.cmet.2024.03.002 38569471 PMC11025492

[39] Mittendorfer B, Kayser BD, Yoshino M, Yoshino J, Watrous JD, Jain M, et al. Heterogeneity in the effect of marked weight loss on metabolic function in women with obesity. JCI Insight. 2023;8(12):e169541. doi: 10.1172/jci.insight.169541 37159276 PMC10371235

[40] Mathis D. Organismal immunometabolism: advances in both directions. Nat Rev Immunol. 2019;19(2):83–4. doi: 10.1038/s41577-018-0118-z 30635620

[41] Schleh MW, Caslin HL, Garcia JN, Mashayekhi M, Srivastava G, Bradley AB, et al. Metaflammation in obesity and its therapeutic targeting. Sci Transl Med. 2023;15(723):eadf9382. doi: 10.1126/scitranslmed.adf9382 37992150 PMC10847980

[42] Ofei F, Hurel S, Newkirk J, Sopwith M, Taylor R. Effects of an engineered human anti-TNF-α antibody (CDP571) on insulin sensitivity and glycemic control in patients with NIDDM. Diabetes. 1996;45(7):881–5. doi: 10.2337/diab.45.7.881 8666137

[43] Dominguez H, Storgaard H, Rask-Madsen C, Steffen Hermann T, Ihlemann N, Baunbjerg Nielsen D, et al. Metabolic and vascular effects of tumor necrosis factor-alpha blockade with etanercept in obese patients with type 2 diabetes. J Vasc Res. 2005;42(6):517–25. doi: 10.1159/000088261 16155368

[44] Bernstein LE, Berry J, Kim S, Canavan B, Grinspoon SK. Effects of etanercept in patients with the metabolic syndrome. Arch Intern Med. 2006;166(8):902–8. doi: 10.1001/archinte.166.8.902 16636217 PMC3196549

[45] Wascher TC, Lindeman JHN, Sourij H, Kooistra T, Pacini G, Roden M. Chronic TNF-α neutralization does not improve insulin resistance or endothelial function in “healthy” men with metabolic syndrome. Mol Med. 2011;17(3–4):189–93. doi: 10.2119/molmed.2010.00221 21103669 PMC3060990

[46] Paquot N, Castillo MJ, Lefèbvre PJ, Scheen AJ. No increased insulin sensitivity after a single intravenous administration of a recombinant human tumor necrosis factor receptor: Fc fusion protein in obese insulin-resistant patients. J Clin Endocrinol Metab. 2000;85(3):1316–9. doi: 10.1210/jcem.85.3.6417 10720082

[47] Oral EA, Reilly SM, Gomez AV, Meral R, Butz L, Ajluni N, et al. Inhibition of IKKɛ and TBK1 improves glucose control in a subset of patients with type 2 diabetes. Cell Metab. 2017;26(1):157-170.e7. doi: 10.1016/j.cmet.2017.06.006 28683283 PMC5663294

[48] Lee YS, Olefsky J. Chronic tissue inflammation and metabolic disease. Genes Dev. 2021;35(5–6):307–28. doi: 10.1101/gad.346312.120 33649162 PMC7919414

[49] Lee YS, Li P, Huh JY, Hwang IJ, Lu M, Kim JI, et al. Inflammation is necessary for long-term but not short-term high-fat diet-induced insulin resistance. Diabetes. 2011;60(10):2474–83. doi: 10.2337/db11-0194 21911747 PMC3178297

[50] Boden G, Homko C, Barrero CA, Stein TP, Chen X, Cheung P, et al. Excessive caloric intake acutely causes oxidative stress, GLUT4 carbonylation, and insulin resistance in healthy men. Sci Transl Med. 2015;7(304):304re7. doi: 10.1126/scitranslmed.aac4765 26355033 PMC5600191

[51] Zhu Q, An YA, Kim M, Zhang Z, Zhao S, Zhu Y, et al. Suppressing adipocyte inflammation promotes insulin resistance in mice. Mol Metab. 2020;39:101010. doi: 10.1016/j.molmet.2020.101010 32408016 PMC7272509

[52] Chen Q, Lai SM, Xu S, Tan Y, Leong K, Liu D, et al. Resident macrophages restrain pathological adipose tissue remodeling and protect vascular integrity in obese mice. EMBO Rep. 2021;22(8):e52835. doi: 10.15252/embr.202152835 34196465 PMC8339675

[53] Jaitin DA, Adlung L, Thaiss CA, Weiner A, Li B, Descamps H, et al. Lipid-associated macrophages control metabolic homeostasis in a Trem2-dependent manner. Cell. 2019;178(3):686-698.e14. doi: 10.1016/j.cell.2019.05.054 31257031 PMC7068689

[54] Wernstedt Asterholm I, Tao C, Morley TS, Wang QA, Delgado-Lopez F, Wang ZV, et al. Adipocyte inflammation is essential for healthy adipose tissue expansion and remodeling. Cell Metab. 2014;20(1):103–18. doi: 10.1016/j.cmet.2014.05.005 24930973 PMC4079756

[55] Tao C, Holland WL, Wang QA, Shao M, Jia L, Sun K, et al. Short-term versus long-term effects of adipocyte toll-like receptor 4 activation on insulin resistance in male mice. Endocrinology. 2017;158(5):1260–70. doi: 10.1210/en.2017-00024 28323977 PMC5460839

[56] Zhu Q, An YA, Kim M, Zhang Z, Zhao S, Zhu Y, et al. Suppressing adipocyte inflammation promotes insulin resistance in mice. Mol Metab. 2020;39:101010. doi: 10.1016/j.molmet.2020.101010 32408016 PMC7272509

[57] Shi H, Kokoeva MV, Inouye K, Tzameli I, Yin H, Flier JS. TLR4 links innate immunity and fatty acid-induced insulin resistance. J Clin Invest. 2006;116(11):3015–25. doi: 10.1172/JCI28898 17053832 PMC1616196

[58] Kim F, Pham M, Luttrell I, Bannerman DD, Tupper J, Thaler J, et al. Toll-like receptor-4 mediates vascular inflammation and insulin resistance in diet-induced obesity. Circ Res. 2007;100(11):1589–96. doi: 10.1161/CIRCRESAHA.106.142851 17478729

[59] Saberi M, Woods N-B, de Luca C, Schenk S, Lu JC, Bandyopadhyay G, et al. Hematopoietic cell-specific deletion of toll-like receptor 4 ameliorates hepatic and adipose tissue insulin resistance in high-fat-fed mice. Cell Metab. 2009;10(5):419–29. doi: 10.1016/j.cmet.2009.09.006 19883619 PMC2790319

[60] Davis JE, Gabler NK, Walker-Daniels J, Spurlock ME. Tlr-4 deficiency selectively protects against obesity induced by diets high in saturated fat. Obesity (Silver Spring). 2008;16(6):1248–55. doi: 10.1038/oby.2008.210 18421279

[61] Roncon-Albuquerque R Jr, Moreira-Rodrigues M, Faria B, Ferreira AP, Cerqueira C, Lourenço AP, et al. Attenuation of the cardiovascular and metabolic complications of obesity in CD14 knockout mice. Life Sci. 2008;83(13–14):502–10. doi: 10.1016/j.lfs.2008.07.021 18761356

[62] Crewe C, An YA, Scherer PE. The ominous triad of adipose tissue dysfunction: inflammation, fibrosis, and impaired angiogenesis. J Clin Invest. 2017;127(1):74–82. doi: 10.1172/JCI88883 28045400 PMC5199684

[63] Yuan M, Konstantopoulos N, Lee J, Hansen L, Li ZW, Karin M, et al. Reversal of obesity- and diet-induced insulin resistance with salicylates or targeted disruption of Ikkbeta. Science. 2001;293(5535):1673–7. doi: 10.1126/science.1061620 11533494

[64] Kim JK, Kim YJ, Fillmore JJ, Chen Y, Moore I, Lee J, et al. Prevention of fat-induced insulin resistance by salicylate. J Clin Invest. 2001;108(3):437–46. doi: 10.1172/JCI11559 11489937 PMC209353

[65] Arkan MC, Hevener AL, Greten FR, Maeda S, Li Z-W, Long JM, et al. IKK-beta links inflammation to obesity-induced insulin resistance. Nat Med. 2005;11(2):191–8. doi: 10.1038/nm1185 15685170

[66] Cai D, Yuan M, Frantz DF, Melendez PA, Hansen L, Lee J, et al. Local and systemic insulin resistance resulting from hepatic activation of IKK-beta and NF-κB. Nat Med. 2005;11(2):183–90. doi: 10.1038/nm1166 15685173 PMC1440292

[67] Liu J, Ibi D, Taniguchi K, Lee J, Herrema H, Akosman B, et al. Inflammation improves glucose homeostasis through IKKβ-XBP1s interaction. Cell. 2016;167(4):1052-1066.e18. doi: 10.1016/j.cell.2016.10.015 27814504 PMC5908236

[68] Kwon H, Laurent S, Tang Y, Zong H, Vemulapalli P, Pessin JE. Adipocyte-specific IKKβ signaling suppresses adipose tissue inflammation through an IL-13-dependent paracrine feedback pathway. Cell Rep. 2014;9(5):1574–83. doi: 10.1016/j.celrep.2014.10.068 25466256 PMC4268106

[69] Fuchs A, Samovski D, Smith GI, Cifarelli V, Farabi SS, Yoshino J, et al. Associations among adipose tissue immunology, inflammation, exosomes and insulin sensitivity in people with obesity and nonalcoholic fatty liver disease. Gastroenterology. 2021;161(3):968-981.e12. doi: 10.1053/j.gastro.2021.05.008 34004161 PMC8900214

[70] Fazeli PK, Zhang Y, O’Keefe J, Pesaresi T, Lun M, Lawney B, et al. Prolonged fasting drives a program of metabolic inflammation in human adipose tissue. Mol Metab. 2020;42:101082. doi: 10.1016/j.molmet.2020.101082 32992039 PMC7554650

[71] Kosteli A, Sugaru E, Haemmerle G, Martin JF, Lei J, Zechner R, et al. Weight loss and lipolysis promote a dynamic immune response in murine adipose tissue. J Clin Invest. 2010;120(10):3466–79. doi: 10.1172/JCI42845 20877011 PMC2947229

[72] Li P, Lu M, Nguyen MTA, Bae EJ, Chapman J, Feng D, et al. Functional heterogeneity of CD11c-positive adipose tissue macrophages in diet-induced obese mice. J Biol Chem. 2010;285(20):15333–45. doi: 10.1074/jbc.M110.100263 20308074 PMC2865288

[73] Magkos F, Fraterrigo G, Yoshino J, Luecking C, Kirbach K, Kelly SC, et al. Effects of moderate and subsequent progressive weight loss on metabolic function and adipose tissue biology in humans with obesity. Cell Metab. 2016;23(4):591–601. doi: 10.1016/j.cmet.2016.02.005 26916363 PMC4833627

[74] Villarreal-Calderon JR, Cuellar-Tamez R, Castillo EC, Luna-Ceron E, García-Rivas G, Elizondo-Montemayor L. Metabolic shift precedes the resolution of inflammation in a cohort of patients undergoing bariatric and metabolic surgery. Sci Rep. 2021;11(1):12127. doi: 10.1038/s41598-021-91393-y 34108550 PMC8190106

[75] Espinosa De Ycaza AE, Søndergaard E, Morgan-Bathke M, Lytle K, Delivanis DA, Ramos P, et al. Adipose tissue inflammation is not related to adipose insulin resistance in humans. Diabetes. 2022;71(3):381–93. doi: 10.2337/db21-0609 34857544 PMC8893944

[76] Cottam MA, Caslin HL, Winn NC, Hasty AH. Multiomics reveals persistence of obesity-associated immune cell phenotypes in adipose tissue during weight loss and weight regain in mice. Nat Commun. 2022;13(1):2950. doi: 10.1038/s41467-022-30646-4 35618862 PMC9135744

[77] Rosen ED, Kajimura S. Is it time to rethink the relationship between adipose inflammation and insulin resistance? J Clin Invest. 2024;134(17):e184663. doi: 10.1172/JCI184663 39225103 PMC11364379

[78] Scherer PE. The multifaceted roles of adipose tissue-therapeutic targets for diabetes and beyond: the 2015 Banting Lecture. Diabetes. 2016;65(6):1452–61. doi: 10.2337/db16-0339 27222389 PMC4878420

[79] Jeelani I, Franco da Cunha F, Rohm TV, Nasamran CA, Wang L, Moon J-S, et al. CCL26 and CXCL12 preserve insulin-sensitizing macrophages in subcutaneous adipose tissue in obesity. Cell Rep. 2025;44(10):116450. doi: 10.1016/j.celrep.2025.116450 41100258 PMC12587491

[80] Wiedemann MSF, Wueest S, Item F, Schoenle EJ, Konrad D. Adipose tissue inflammation contributes to short-term high-fat diet-induced hepatic insulin resistance. Am J Physiol Endocrinol Metab. 2013;305(3):E388-95. doi: 10.1152/ajpendo.00179.2013 23736545

[81] Ji Y, Sun S, Xia S, Yang L, Li X, Qi L. Short term high fat diet challenge promotes alternative macrophage polarization in adipose tissue via natural killer T cells and interleukin-4. J Biol Chem. 2012;287(29):24378–86. doi: 10.1074/jbc.M112.371807 22645141 PMC3397864

[82] Sekimoto R, Fukuda S, Maeda N, Tsushima Y, Matsuda K, Mori T, et al. Visualized macrophage dynamics and significance of S100A8 in obese fat. Proc Natl Acad Sci U S A. 2015;112(16):E2058-66. doi: 10.1073/pnas.1409480112 25848057 PMC4413348

[83] Sabio G, Das M, Mora A, Zhang Z, Jun JY, Ko HJ, et al. A stress signaling pathway in adipose tissue regulates hepatic insulin resistance. Science. 2008;322(5907):1539–43. doi: 10.1126/science.1160794 19056984 PMC2643026

[84] Lee YS, Kim J-W, Osborne O, Oh DY, Sasik R, Schenk S, et al. Increased adipocyte O2 consumption triggers HIF-1α, causing inflammation and insulin resistance in obesity. Cell. 2014;157(6):1339–52. doi: 10.1016/j.cell.2014.05.012 24906151 PMC4114226

[85] Lee KY, Gesta S, Boucher J, Wang XL, Kahn CR. The differential role of Hif1β/Arnt and the hypoxic response in adipose function, fibrosis, and inflammation. Cell Metab. 2011;14(4):491–503. doi: 10.1016/j.cmet.2011.08.006 21982709 PMC3206000

[86] Jiang C, Qu A, Matsubara T, Chanturiya T, Jou W, Gavrilova O, et al. Disruption of hypoxia-inducible factor 1 in adipocytes improves insulin sensitivity and decreases adiposity in high-fat diet-fed mice. Diabetes. 2011;60(10):2484–95. doi: 10.2337/db11-0174 21873554 PMC3178277

[87] Kanda H, Tateya S, Tamori Y, Kotani K, Hiasa K, Kitazawa R, et al. MCP-1 contributes to macrophage infiltration into adipose tissue, insulin resistance, and hepatic steatosis in obesity. J Clin Invest. 2006;116(6):1494–505. doi: 10.1172/JCI26498 16691291 PMC1459069

[88] Sun K, Halberg N, Khan M, Magalang UJ, Scherer PE. Selective inhibition of hypoxia-inducible factor 1α ameliorates adipose tissue dysfunction. Mol Cell Biol. 2013;33(5):904–17. doi: 10.1128/MCB.00951-12 23249949 PMC3623075

[89] Lee YS, Riopel M, Cabrales P, Bandyopadhyay GK. Hepatocyte-specific HIF-1α ablation improves obesity-induced glucose intolerance by reducing first-pass GLP-1 degradation. Sci Adv. 2019;5(7):eaaw4176. doi: 10.1126/sciadv.aaw4176 31281892 PMC6609217

[90] Seo JB, Riopel M, Cabrales P, Huh JY, Bandyopadhyay GK, Andreyev AY, et al. Knockdown of Ant2 reduces adipocyte hypoxia and improves insulin resistance in obesity. Nat Metab. 2019;1(1):86–97. doi: 10.1038/s42255-018-0003-x 31528845 PMC6746433

[91] Hosogai N, Fukuhara A, Oshima K, Miyata Y, Tanaka S, Segawa K, et al. Adipose tissue hypoxia in obesity and its impact on adipocytokine dysregulation. Diabetes. 2007;56(4):901–11. doi: 10.2337/db06-0911 17395738

[92] Pasarica M, Rood J, Ravussin E, Schwarz J-M, Smith SR, Redman LM. Reduced oxygenation in human obese adipose tissue is associated with impaired insulin suppression of lipolysis. J Clin Endocrinol Metab. 2010;95(8):4052–5. doi: 10.1210/jc.2009-2377 20466783 PMC2913036

[93] Sun K, Wernstedt Asterholm I, Kusminski CM, Bueno AC, Wang ZV, Pollard JW, et al. Dichotomous effects of VEGF-A on adipose tissue dysfunction. Proc Natl Acad Sci U S A. 2012;109(15):5874–9. doi: 10.1073/pnas.1200447109 22451920 PMC3326476

[94] Robciuc MR, Kivelä R, Williams IM, de Boer JF, van Dijk TH, Elamaa H, et al. VEGFB/VEGFR1-induced expansion of adipose vasculature counteracts obesity and related metabolic complications. Cell Metab. 2016;23(4):712–24. doi: 10.1016/j.cmet.2016.03.004 27076080 PMC5898626

[95] Elias I, Franckhauser S, Ferré T, Vilà L, Tafuro S, Muñoz S, et al. Adipose tissue overexpression of vascular endothelial growth factor protects against diet-induced obesity and insulin resistance. Diabetes. 2012;61(7):1801–13. doi: 10.2337/db11-0832 22522611 PMC3379662

[96] Sung H-K, Doh K-O, Son JE, Park JG, Bae Y, Choi S, et al. Adipose vascular endothelial growth factor regulates metabolic homeostasis through angiogenesis. Cell Metab. 2013;17(1):61–72. doi: 10.1016/j.cmet.2012.12.010 23312284

[97] Li P, Oh DY, Bandyopadhyay G, Lagakos WS, Talukdar S, Osborn O, et al. LTB4 promotes insulin resistance in obese mice by acting on macrophages, hepatocytes and myocytes. Nat Med. 2015;21(3):239–47. doi: 10.1038/nm.3800 25706874 PMC4429798

[98] Lumeng CN, Bodzin JL, Saltiel AR. Obesity induces a phenotypic switch in adipose tissue macrophage polarization. J Clin Invest. 2007;117(1):175–84. doi: 10.1172/JCI29881 17200717 PMC1716210

[99] Amano SU, Cohen JL, Vangala P, Tencerova M, Nicoloro SM, Yawe JC, et al. Local proliferation of macrophages contributes to obesity-associated adipose tissue inflammation. Cell Metab. 2014;19(1):162–71. doi: 10.1016/j.cmet.2013.11.017 24374218 PMC3931314

[100] Jo W, Kim M, Oh J, Kim C-S, Park C, Yoon S, et al. MicroRNA-29 ameliorates fibro-inflammation and insulin resistance in HIF1α-deficient obese adipose tissue by inhibiting endotrophin generation. Diabetes. 2022;71(8):1746–62. doi: 10.2337/db21-0801 35167651

[101] Halberg N, Khan T, Trujillo ME, Wernstedt-Asterholm I, Attie AD, Sherwani S, et al. Hypoxia-inducible factor 1α induces fibrosis and insulin resistance in white adipose tissue. Mol Cell Biol. 2009;29(16):4467–83. doi: 10.1128/MCB.00192-09 19546236 PMC2725728

[102] Sun K, Park J, Gupta OT, Holland WL, Auerbach P, Zhang N, et al. Endotrophin triggers adipose tissue fibrosis and metabolic dysfunction. Nat Commun. 2014;5:3485. doi: 10.1038/ncomms4485 24647224 PMC4076823

[103] Abram CL, Roberge GL, Hu Y, Lowell CA. Comparative analysis of the efficiency and specificity of myeloid-Cre deleting strains using ROSA-EYFP reporter mice. J Immunol Methods. 2014;408:89–100. doi: 10.1016/j.jim.2014.05.009 24857755 PMC4105345

[104] Kihira Y, Miyake M, Hirata M, Hoshina Y, Kato K, Shirakawa H, et al. Deletion of hypoxia-inducible factor-1α in adipocytes enhances glucagon-like peptide-1 secretion and reduces adipose tissue inflammation. PLoS One. 2014;9(4):e93856. doi: 10.1371/journal.pone.0093856 24705496 PMC3976326

[105] García-Martín R, Alexaki VI, Qin N, Rubín de Celis MF, Economopoulou M, Ziogas A, et al. Adipocyte-specific hypoxia-inducible factor 2α deficiency exacerbates obesity-induced brown adipose tissue dysfunction and metabolic dysregulation. Mol Cell Biol. 2015;36(3):376–93. doi: 10.1128/MCB.00430-15 26572826 PMC4719429

[106] Han JS, Jeon YG, Oh M, Lee G, Nahmgoong H, Han SM, et al. Adipocyte HIF2α functions as a thermostat via PKA Cα regulation in beige adipocytes. Nat Commun. 2022;13(1):3268. doi: 10.1038/s41467-022-30925-0 35672324 PMC9174489

[107] Jeelani I, Moon J-S, da Cunha FF, Nasamran CA, Jeon S, Zhang X, et al. HIF-2α drives hepatic Kupffer cell death and proinflammatory recruited macrophage activation in nonalcoholic steatohepatitis. Sci Transl Med. 2024;16(764):eadi0284. doi: 10.1126/scitranslmed.adi0284 39259813 PMC11665927

[108] Ye J, Gao Z, Yin J, He Q. Hypoxia is a potential risk factor for chronic inflammation and adiponectin reduction in adipose tissue of *ob/ob* and dietary obese mice. Am J Physiol Endocrinol Metab. 2007;293(4):E1118-28. doi: 10.1152/ajpendo.00435.2007 17666485

[109] Nishimura S, Manabe I, Nagasaki M, Seo K, Yamashita H, Hosoya Y, et al. In vivo imaging in mice reveals local cell dynamics and inflammation in obese adipose tissue. J Clin Invest. 2008;118(2):710–21. doi: 10.1172/JCI33328 18202748 PMC2200301

[110] Smith GI, Mittendorfer B, Klein S. Metabolically healthy obesity: facts and fantasies. J Clin Invest. 2019;129(10):3978–89. doi: 10.1172/JCI129186 31524630 PMC6763224

[111] Pasarica M, Sereda OR, Redman LM, Albarado DC, Hymel DT, Roan LE, et al. Reduced adipose tissue oxygenation in human obesity: evidence for rarefaction, macrophage chemotaxis, and inflammation without an angiogenic response. Diabetes. 2009;58(3):718–25. doi: 10.2337/db08-1098 19074987 PMC2646071

[112] Lawler HM, Underkofler CM, Kern PA, Erickson C, Bredbeck B, Rasouli N. Adipose tissue hypoxia, inflammation, and fibrosis in obese insulin-sensitive and obese insulin-resistant subjects. J Clin Endocrinol Metab. 2016;101(4):1422–8. doi: 10.1210/jc.2015-4125 26871994 PMC4880157

[113] Cifarelli V, Beeman SC, Smith GI, Yoshino J, Morozov D, Beals JW, et al. Decreased adipose tissue oxygenation associates with insulin resistance in individuals with obesity. J Clin Invest. 2020;130(12):6688–99. doi: 10.1172/JCI141828 33164985 PMC7685757

[114] Goossens GH, Bizzarri A, Venteclef N, Essers Y, Cleutjens JP, Konings E, et al. Increased adipose tissue oxygen tension in obese compared with lean men is accompanied by insulin resistance, impaired adipose tissue capillarization, and inflammation. Circulation. 2011;124(1):67–76. doi: 10.1161/CIRCULATIONAHA.111.027813 21670228

[115] Goossens GH, Vogel MAA, Vink RG, Mariman EC, van Baak MA, Blaak EE. Adipose tissue oxygenation is associated with insulin sensitivity independently of adiposity in obese men and women. Diabetes Obes Metab. 2018;20(9):2286–90. doi: 10.1111/dom.13329 29687584

[116] Oyadomari S, Harding HP, Zhang Y, Oyadomari M, Ron D. Dephosphorylation of translation initiation factor 2α enhances glucose tolerance and attenuates hepatosteatosis in mice. Cell Metab. 2008;7(6):520–32. doi: 10.1016/j.cmet.2008.04.011 18522833 PMC2474721

[117] Fu S, Yang L, Li P, Hofmann O, Dicker L, Hide W, et al. Aberrant lipid metabolism disrupts calcium homeostasis causing liver endoplasmic reticulum stress in obesity. Nature. 2011;473(7348):528–31. doi: 10.1038/nature09968 21532591 PMC3102791

[118] Hotamisligil GS, Davis RJ. Cell signaling and stress responses. Cold Spring Harb Perspect Biol. 2016;8(10):a006072. doi: 10.1101/cshperspect.a006072 27698029 PMC5046695

[119] Furukawa S, Fujita T, Shimabukuro M, Iwaki M, Yamada Y, Nakajima Y, et al. Increased oxidative stress in obesity and its impact on metabolic syndrome. J Clin Invest. 2004;114(12):1752–61. doi: 10.1172/JCI21625 15599400 PMC535065

[120] Lee YS, Kim AY, Choi JW, Kim M, Yasue S, Son HJ, et al. Dysregulation of adipose glutathione peroxidase 3 in obesity contributes to local and systemic oxidative stress. Mol Endocrinol. 2008;22(9):2176–89. doi: 10.1210/me.2008-0023 18562625 PMC5419460

[121] Park J, Rho HK, Kim KH, Choe SS, Lee YS, Kim JB. Overexpression of glucose-6-phosphate dehydrogenase is associated with lipid dysregulation and insulin resistance in obesity. Mol Cell Biol. 2005;25(12):5146–57. doi: 10.1128/MCB.25.12.5146-5157.2005 15923630 PMC1140588

[122] Perreault M, Marette A. Targeted disruption of inducible nitric oxide synthase protects against obesity-linked insulin resistance in muscle. Nat Med. 2001;7(10):1138–43. doi: 10.1038/nm1001-1138 11590438

[123] Funcke J-B, Scherer PE. Beyond adiponectin and leptin: adipose tissue-derived mediators of inter-organ communication. J Lipid Res. 2019;60(10):1648–84. doi: 10.1194/jlr.R094060 31209153 PMC6795086

[124] Sharma M, Boytard L, Hadi T, Koelwyn G, Simon R, Ouimet M, et al. Enhanced glycolysis and HIF-1α activation in adipose tissue macrophages sustains local and systemic interleukin-1β production in obesity. Sci Rep. 2020;10(1):5555. doi: 10.1038/s41598-020-62272-9 32221369 PMC7101445

[125] Moon J-S, da Cunha FF, Huh JY, Andreyev AY, Lee J, Mahata SK, et al. ANT2 drives proinflammatory macrophage activation in obesity. JCI Insight. 2021;6(20):e147033. doi: 10.1172/jci.insight.147033 34676827 PMC8564915

[126] Takikawa A, Mahmood A, Nawaz A, Kado T, Okabe K, Yamamoto S, et al. HIF-1α in myeloid cells promotes adipose tissue remodeling toward insulin resistance. Diabetes. 2016;65(12):3649–59. doi: 10.2337/db16-0012 27625023

[127] Wang X, de Carvalho Ribeiro M, Iracheta-Vellve A, Lowe P, Ambade A, Satishchandran A, et al. Macrophage-specific hypoxia-inducible factor-1α contributes to impaired autophagic flux in nonalcoholic steatohepatitis. Hepatology. 2019;69(2):545–63. doi: 10.1002/hep.30215 30102772 PMC6351177

[128] Farrugia MA, Pini E, Tran A, Chevalier N, Anty R, Gual P. Incretins and MASLD: at the crossroads of endocrine and hepatic disorders. Curr Obes Rep. 2025;14(1):56. doi: 10.1007/s13679-025-00646-8 40562950 PMC12198343

[129] Tilg H, Targher G. Lessons from incretin-based therapy in MASH and obesity. Nat Rev Gastroenterol Hepatol. 2025;22(8):531–3. doi: 10.1038/s41575-025-01096-2 40579476

[130] Massier L, Chakaroun R, Tabei S, Crane A, Didt KD, Fallmann J, et al. Adipose tissue derived bacteria are associated with inflammation in obesity and type 2 diabetes. Gut. 2020;69(10):1796–806. doi: 10.1136/gutjnl-2019-320118 32317332

[131] Cani PD, Amar J, Iglesias MA, Poggi M, Knauf C, Bastelica D, et al. Metabolic endotoxemia initiates obesity and insulin resistance. Diabetes. 2007;56(7):1761–72. doi: 10.2337/db06-1491 17456850

[132] Chan KL, Tam TH, Boroumand P, Prescott D, Costford SR, Escalante NK, et al. Circulating NOD1 activators and hematopoietic NOD1 contribute to metabolic inflammation and insulin resistance. Cell Rep. 2017;18(10):2415–26. doi: 10.1016/j.celrep.2017.02.027 28273456

[133] Kupczyk D, Bilski R, Kozakiewicz M, Studzińska R, Kędziora-Kornatowska K, Kosmalski T, et al. 11β-HSD as a new target in pharmacotherapy of metabolic diseases. Int J Mol Sci. 2022;23(16):8984. doi: 10.3390/ijms23168984 36012251 PMC9409048

[134] Vieira E, Mirizio GG, Barin GR, de Andrade RV, Nimer NFS, La Sala L. Clock genes, inflammation and the immune system-implications for diabetes, obesity and neurodegenerative diseases. Int J Mol Sci. 2020;21(24):9743. doi: 10.3390/ijms21249743 33371208 PMC7766955

[135] Proto JD, Doran AC, Gusarova G, Yurdagul A Jr, Sozen E, Subramanian M, et al. Regulatory T cells promote macrophage efferocytosis during inflammation resolution. Immunity. 2018;49(4):666-677.e6. doi: 10.1016/j.immuni.2018.07.015 30291029 PMC6192849

[136] Chiang N, Serhan CN. Specialized pro-resolving mediator network: an update on production and actions. Essays Biochem. 2020;64(3):443–62. doi: 10.1042/EBC20200018 32885825 PMC7682745

[137] Neuhofer A, Zeyda M, Mascher D, Itariu BK, Murano I, Leitner L, et al. Impaired local production of proresolving lipid mediators in obesity and 17-HDHA as a potential treatment for obesity-associated inflammation. Diabetes. 2013;62(6):1945–56. doi: 10.2337/db12-0828 23349501 PMC3661630

[138] Barden AE, Mas E, Croft KD, Phillips M, Mori TA. Specialized proresolving lipid mediators in humans with the metabolic syndrome after n-3 fatty acids and aspirin. Am J Clin Nutr. 2015;102(6):1357–64. doi: 10.3945/ajcn.115.116384 26561623

[139] Hong E-G, Ko HJ, Cho Y-R, Kim H-J, Ma Z, Yu TY, et al. Interleukin-10 prevents diet-induced insulin resistance by attenuating macrophage and cytokine response in skeletal muscle. Diabetes. 2009;58(11):2525–35. doi: 10.2337/db08-1261 19690064 PMC2768157

[140] Börgeson E, Johnson AMF, Lee YS, Till A, Syed GH, Ali-Shah ST, et al. Lipoxin A4 attenuates obesity-induced adipose inflammation and associated liver and kidney disease. Cell Metab. 2015;22(1):125–37. doi: 10.1016/j.cmet.2015.05.003 26052006 PMC4584026

[141] Liu X, Tang Y, Luo Y, Gao Y, He L. Role and mechanism of specialized pro-resolving mediators in obesity-associated insulin resistance. Lipids Health Dis. 2024;23(1):234. doi: 10.1186/s12944-024-02207-9 39080624 PMC11290132

[142] Hellmann J, Tang Y, Kosuri M, Bhatnagar A, Spite M. Resolvin D1 decreases adipose tissue macrophage accumulation and improves insulin sensitivity in obese-diabetic mice. FASEB J. 2011;25(7):2399–407. doi: 10.1096/fj.10-178657 21478260 PMC3114534

[143] Clark M, Suur BE, Soták M, Börgeson E. Attenuation of adipose tissue inflammation by pro-resolving lipid mediators. Curr Opin Endocr Metab Res. 2024;36:100539. doi: 10.1016/j.coemr.2024.100539 39619489 PMC11602545

[144] Martínez-Fernández L, González-Muniesa P, Laiglesia LM, Sáinz N, Prieto-Hontoria PL, Escoté X, et al. Maresin 1 improves insulin sensitivity and attenuates adipose tissue inflammation in ob/ob and diet-induced obese mice. FASEB J. 2017;31(5):2135–45. doi: 10.1096/fj.201600859R 28188173

[145] Ozcan U, Ozcan L, Yilmaz E, Düvel K, Sahin M, Manning BD, et al. Loss of the tuberous sclerosis complex tumor suppressors triggers the unfolded protein response to regulate insulin signaling and apoptosis. Mol Cell. 2008;29(5):541–51. doi: 10.1016/j.molcel.2007.12.023 18342602 PMC2361721

[146] Gregor MF, Yang L, Fabbrini E, Mohammed BS, Eagon JC, Hotamisligil GS, et al. Endoplasmic reticulum stress is reduced in tissues of obese subjects after weight loss. Diabetes. 2009;58(3):693–700. doi: 10.2337/db08-1220 19066313 PMC2646068

[147] Sharma NK, Das SK, Mondal AK, Hackney OG, Chu WS, Kern PA, et al. Endoplasmic reticulum stress markers are associated with obesity in nondiabetic subjects. J Clin Endocrinol Metab. 2008;93(11):4532–41. doi: 10.1210/jc.2008-1001 18728164 PMC2582561

[148] Ozcan U, Cao Q, Yilmaz E, Lee A-H, Iwakoshi NN, Ozdelen E, et al. Endoplasmic reticulum stress links obesity, insulin action, and type 2 diabetes. Science. 2004;306(5695):457–61. doi: 10.1126/science.1103160 15486293

[149] Shan B, Wang X, Wu Y, Xu C, Xia Z, Dai J, et al. The metabolic ER stress sensor IRE1α suppresses alternative activation of macrophages and impairs energy expenditure in obesity. Nat Immunol. 2017;18(5):519–29. doi: 10.1038/ni.3709 28346409

[150] Chen Y, Wu Z, Zhao S, Xiang R. Chemical chaperones reduce ER stress and adipose tissue inflammation in high fat diet-induced mouse model of obesity. Sci Rep. 2016;6:27486. doi: 10.1038/srep27486 27271106 PMC4897685

[151] Kawasaki N, Asada R, Saito A, Kanemoto S, Imaizumi K. Obesity-induced endoplasmic reticulum stress causes chronic inflammation in adipose tissue. Sci Rep. 2012;2:799. doi: 10.1038/srep00799 23150771 PMC3495279

[152] Ozcan U, Yilmaz E, Ozcan L, Furuhashi M, Vaillancourt E, Smith RO, et al. Chemical chaperones reduce ER stress and restore glucose homeostasis in a mouse model of type 2 diabetes. Science. 2006;313(5790):1137–40. doi: 10.1126/science.1128294 16931765 PMC4741373

[153] Xia W, Veeragandham P, Cao Y, Xu Y, Rhyne TE, Qian J, et al. Obesity causes mitochondrial fragmentation and dysfunction in white adipocytes due to RalA activation. Nat Metab. 2024;6(2):273–89. doi: 10.1038/s42255-024-00978-0 38286821 PMC10896723

[154] Krauss S, Zhang C-Y, Scorrano L, Dalgaard LT, St-Pierre J, Grey ST, et al. Superoxide-mediated activation of uncoupling protein 2 causes pancreatic beta cell dysfunction. J Clin Invest. 2003;112(12):1831–42. doi: 10.1172/JCI19774 14679178 PMC297000

[155] Moon J-S, Riopel M, Seo JB, Herrero-Aguayo V, Isaac R, Lee YS. HIF-2α Preserves mitochondrial activity and glucose sensing in compensating β-Cells in obesity. Diabetes. 2022;71(7):1508–24. doi: 10.2337/db21-0736 35472707 PMC9233300

[156] Ham M, Choe SS, Shin KC, Choi G, Kim J-W, Noh J-R, et al. Glucose-6-phosphate dehydrogenase deficiency improves insulin resistance with reduced adipose tissue inflammation in obesity. Diabetes. 2016;65(9):2624–38. doi: 10.2337/db16-0060 27284106

[157] Den Hartigh LJ, Omer M, Goodspeed L, Wang S, Wietecha T, O’Brien KD, et al. Adipocyte-specific deficiency of NADPH oxidase 4 delays the onset of insulin resistance and attenuates adipose tissue inflammation in obesity. Arterioscler Thromb Vasc Biol. 2017;37(3):466–75. doi: 10.1161/ATVBAHA.116.308749 28062496 PMC5323321

[158] Kaneto H, Kajimoto Y, Miyagawa J, Matsuoka T, Fujitani Y, Umayahara Y, et al. Beneficial effects of antioxidants in diabetes: possible protection of pancreatic beta-cells against glucose toxicity. Diabetes. 1999;48(12):2398–406. doi: 10.2337/diabetes.48.12.2398 10580429

[159] Feng D, Tang Y, Kwon H, Zong H, Hawkins M, Kitsis RN, et al. High-fat diet-induced adipocyte cell death occurs through a cyclophilin D intrinsic signaling pathway independent of adipose tissue inflammation. Diabetes. 2011;60(8):2134–43. doi: 10.2337/db10-1411 21734017 PMC3142076

[160] Murano I, Barbatelli G, Parisani V, Latini C, Muzzonigro G, Castellucci M, et al. Dead adipocytes, detected as crown-like structures, are prevalent in visceral fat depots of genetically obese mice. J Lipid Res. 2008;49(7):1562–8. doi: 10.1194/jlr.M800019-JLR200 18390487

[161] Cinti S, Mitchell G, Barbatelli G, Murano I, Ceresi E, Faloia E, et al. Adipocyte death defines macrophage localization and function in adipose tissue of obese mice and humans. J Lipid Res. 2005;46(11):2347–55. doi: 10.1194/jlr.M500294-JLR200 16150820

[162] Alkhouri N, Gornicka A, Berk MP, Thapaliya S, Dixon LJ, Kashyap S, et al. Adipocyte apoptosis, a link between obesity, insulin resistance, and hepatic steatosis. J Biol Chem. 2010;285(5):3428–38. doi: 10.1074/jbc.M109.074252 19940134 PMC2823448

[163] Giordano A, Murano I, Mondini E, Perugini J, Smorlesi A, Severi I, et al. Obese adipocytes show ultrastructural features of stressed cells and die of pyroptosis. J Lipid Res. 2013;54(9):2423–36. doi: 10.1194/jlr.M038638 23836106 PMC3735940

[164] Tao Y, Zang J, Wang T, Song P, Zhou Z, Li H, et al. Obesity-associated macrophages dictate adipose stem cell ferroptosis and visceral fat dysfunction by propagating mitochondrial fragmentation. Nat Commun. 2025;16(1):7564. doi: 10.1038/s41467-025-62690-1 40813577 PMC12354832

[165] Palmer AK, Xu M, Zhu Y, Pirtskhalava T, Weivoda MM, Hachfeld CM, et al. Targeting senescent cells alleviates obesity-induced metabolic dysfunction. Aging Cell. 2019;18(3):e12950. doi: 10.1111/acel.12950 30907060 PMC6516193

[166] Lee YS, Wollam J, Olefsky JM. An integrated view of immunometabolism. Cell. 2018;172(1–2):22–40. doi: 10.1016/j.cell.2017.12.025 29328913 PMC8451723

[167] Corvera S, Gealekman O. Adipose tissue angiogenesis: impact on obesity and type-2 diabetes. Biochim Biophys Acta. 2014;1842(3):463–72. doi: 10.1016/j.bbadis.2013.06.003 23770388 PMC3844681

[168] Winn NC, Volk KM, Hasty AH. Regulation of tissue iron homeostasis: the macrophage “ferrostat”. JCI Insight. 2020;5(2):e132964. doi: 10.1172/jci.insight.132964 31996481 PMC7098718

[169] Reilly SM, Saltiel AR. Adapting to obesity with adipose tissue inflammation. Nat Rev Endocrinol. 2017;13(11):633–43. doi: 10.1038/nrendo.2017.90 28799554

[170] Brandao BB, Lino M, Kahn CR. Extracellular miRNAs as mediators of obesity-associated disease. J Physiol. 2022;600(5):1155–69. doi: 10.1113/JP280910 34392542 PMC8845532

[171] Hinte LC, Castellano-Castillo D, Ghosh A, Melrose K, Gasser E, Noé F, et al. Adipose tissue retains an epigenetic memory of obesity after weight loss. Nature. 2024;636(8042):457–65. doi: 10.1038/s41586-024-08165-7 39558077 PMC11634781

[172] Chaurasia B, Summers SA. Ceramides in metabolism: key lipotoxic players. Annu Rev Physiol. 2021;83:303–30. doi: 10.1146/annurev-physiol-031620-093815 33158378 PMC7905841

[173] Chavakis T, Alexaki VI, Ferrante AW Jr. Macrophage function in adipose tissue homeostasis and metabolic inflammation. Nat Immunol. 2023;24(5):757–66. doi: 10.1038/s41590-023-01479-0 37012544

[174] Ying W, Fu W, Lee YS, Olefsky JM. The role of macrophages in obesity-associated islet inflammation and β-cell abnormalities. Nat Rev Endocrinol. 2020;16(2):81–90. doi: 10.1038/s41574-019-0286-3 31836875 PMC8315273

[175] Ying W, Lee YS, Dong Y, Seidman JS, Yang M, Isaac R, et al. Expansion of islet-resident macrophages leads to inflammation affecting β cell proliferation and function in obesity. Cell Metab. 2019;29(2):457-474.e5. doi: 10.1016/j.cmet.2018.12.003 30595478 PMC6701710

[176] Zeyda M, Farmer D, Todoric J, Aszmann O, Speiser M, Györi G, et al. Human adipose tissue macrophages are of an anti-inflammatory phenotype but capable of excessive pro-inflammatory mediator production. Int J Obes (Lond). 2007;31(9):1420–8. doi: 10.1038/sj.ijo.0803632 17593905

[177] Li P, Liu S, Lu M, Bandyopadhyay G, Oh D, Imamura T, et al. Hematopoietic-derived galectin-3 causes cellular and systemic insulin resistance. Cell. 2016;167(4):973-984.e12. doi: 10.1016/j.cell.2016.10.025 27814523 PMC5179329

[178] Ying W, Riopel M, Bandyopadhyay G, Dong Y, Birmingham A, Seo JB, et al. Adipose tissue macrophage-derived exosomal miRNAs can modulate in vivo and in vitro insulin sensitivity. Cell. 2017;171(2):372–84 e12. Epub 20170921. doi: 10.1016/j.cell.2017.08.035 28942920

[179] Bu L, Gao M, Qu S, Liu D. Intraperitoneal injection of clodronate liposomes eliminates visceral adipose macrophages and blocks high-fat diet-induced weight gain and development of insulin resistance. AAPS J. 2013;15(4):1001–11. doi: 10.1208/s12248-013-9501-7 23821353 PMC3787235

[180] Feng B, Jiao P, Nie Y, Kim T, Jun D, van Rooijen N, et al. Clodronate liposomes improve metabolic profile and reduce visceral adipose macrophage content in diet-induced obese mice. PLoS One. 2011;6(9):e24358. doi: 10.1371/journal.pone.0024358 21931688 PMC3171445

[181] Huang W, Metlakunta A, Dedousis N, Zhang P, Sipula I, Dube JJ, et al. Depletion of liver Kupffer cells prevents the development of diet-induced hepatic steatosis and insulin resistance. Diabetes. 2010;59(2):347–57. doi: 10.2337/db09-0016 19934001 PMC2809951

[182] Han MS, Jung DY, Morel C, Lakhani SA, Kim JK, Flavell RA, et al. JNK expression by macrophages promotes obesity-induced insulin resistance and inflammation. Science. 2013;339(6116):218–22. doi: 10.1126/science.1227568 23223452 PMC3835653

[183] Dalmas E, Toubal A, Alzaid F, Blazek K, Eames HL, Lebozec K, et al. Irf5 deficiency in macrophages promotes beneficial adipose tissue expansion and insulin sensitivity during obesity. Nat Med. 2015;21(6):610–8. doi: 10.1038/nm.3829 25939064

[184] Tamura Y, Sugimoto M, Murayama T, Ueda Y, Kanamori H, Ono K, et al. Inhibition of CCR2 ameliorates insulin resistance and hepatic steatosis in db/db mice. Arterioscler Thromb Vasc Biol. 2008;28(12):2195–201. doi: 10.1161/ATVBAHA.108.168633 18818420

[185] Lumeng CN, Deyoung SM, Bodzin JL, Saltiel AR. Increased inflammatory properties of adipose tissue macrophages recruited during diet-induced obesity. Diabetes. 2007;56(1):16–23. doi: 10.2337/db06-1076 17192460

[186] Cox N, Crozet L, Holtman IR, Loyher P-L, Lazarov T, White JB, et al. Diet-regulated production of PDGFcc by macrophages controls energy storage. Science. 2021;373(6550):eabe9383. doi: 10.1126/science.abe9383 34210853 PMC9558257

[187] Magalhaes MS, Smith P, Portman JR, Jackson-Jones LH, Bain CC, Ramachandran P, et al. Role of Tim4 in the regulation of ABCA1+ adipose tissue macrophages and post-prandial cholesterol levels. Nat Commun. 2021;12(1):4434. doi: 10.1038/s41467-021-24684-7 34290249 PMC8295389

[188] Orr JS, Kennedy A, Anderson-Baucum EK, Webb CD, Fordahl SC, Erikson KM, et al. Obesity alters adipose tissue macrophage iron content and tissue iron distribution. Diabetes. 2014;63(2):421–32. doi: 10.2337/db13-0213 24130337 PMC3900546

[189] Hubler MJ, Erikson KM, Kennedy AJ, Hasty AH. MFehi adipose tissue macrophages compensate for tissue iron perturbations in mice. Am J Physiol Cell Physiol. 2018;315(3):C319–29. doi: 10.1152/ajpcell.00103.2018 29768045 PMC6171041

[190] Jeelani I, da Cunha FF, Rohm TV, Nasamran CA, Moon J-S, Wang L, et al. CCL26 and CXCL12 promote release of insulin-sensitizing adipose tissue macrophage sEVs from subcutaneous adipose tissue in obesity. bioRxiv. 2025;:2025.08.20.670151. doi: 10.1101/2025.08.20.670151 40894782 PMC12393446

[191] Kanda H, Tateya S, Tamori Y, Kotani K, Hiasa K, Kitazawa R, et al. MCP-1 contributes to macrophage infiltration into adipose tissue, insulin resistance, and hepatic steatosis in obesity. J Clin Invest. 2006;116(6):1494–505. doi: 10.1172/JCI26498 16691291 PMC1459069

[192] Shimizu I, Yoshida Y, Moriya J, Nojima A, Uemura A, Kobayashi Y, et al. Semaphorin3E-induced inflammation contributes to insulin resistance in dietary obesity. Cell Metab. 2013;18(4):491–504. doi: 10.1016/j.cmet.2013.09.001 24093674

[193] Oh DY, Morinaga H, Talukdar S, Bae EJ, Olefsky JM. Increased macrophage migration into adipose tissue in obese mice. Diabetes. 2012;61(2):346–54. doi: 10.2337/db11-0860 22190646 PMC3266418

[194] Ramkhelawon B, Hennessy EJ, Ménager M, Ray TD, Sheedy FJ, Hutchison S, et al. Netrin-1 promotes adipose tissue macrophage retention and insulin resistance in obesity. Nat Med. 2014;20(4):377–84. doi: 10.1038/nm.3467 24584118 PMC3981930

[195] Wanschel A, Seibert T, Hewing B, Ramkhelawon B, Ray TD, van Gils JM, et al. Neuroimmune guidance cue Semaphorin 3E is expressed in atherosclerotic plaques and regulates macrophage retention. Arterioscler Thromb Vasc Biol. 2013;33(5):886–93. doi: 10.1161/ATVBAHA.112.300941 23430613 PMC3647027

[196] Zheng C, Yang Q, Cao J, Xie N, Liu K, Shou P, et al. Local proliferation initiates macrophage accumulation in adipose tissue during obesity. Cell Death Dis. 2016;7(3):e2167. doi: 10.1038/cddis.2016.54 27031964 PMC4823955

[197] Patsouris D, Li P-P, Thapar D, Chapman J, Olefsky JM, Neels JG. Ablation of CD11c-positive cells normalizes insulin sensitivity in obese insulin resistant animals. Cell Metab. 2008;8(4):301–9. doi: 10.1016/j.cmet.2008.08.015 18840360 PMC2630775

[198] Mills CD, Kincaid K, Alt JM, Heilman MJ, Hill AM. M-1/M-2 macrophages and the Th1/Th2 paradigm. J Immunol. 2000;164(12):6166–73. doi: 10.4049/jimmunol.164.12.6166 10843666

[199] Koelwyn GJ, Corr EM, Erbay E, Moore KJ. Regulation of macrophage immunometabolism in atherosclerosis. Nat Immunol. 2018;19(6):526–37. doi: 10.1038/s41590-018-0113-3 29777212 PMC6314674

[200] Jakubzick CV, Randolph GJ, Henson PM. Monocyte differentiation and antigen-presenting functions. Nat Rev Immunol. 2017;17(6):349–62. doi: 10.1038/nri.2017.28 28436425

[201] Li C, Menoret A, Farragher C, Ouyang Z, Bonin C, Holvoet P, et al. Single cell transcriptomics based-MacSpectrum reveals novel macrophage activation signatures in diseases. JCI Insight. 2019;5(10):e126453. doi: 10.1172/jci.insight.126453 30990466 PMC6542613

[202] Kratz M, Coats BR, Hisert KB, Hagman D, Mutskov V, Peris E, et al. Metabolic dysfunction drives a mechanistically distinct proinflammatory phenotype in adipose tissue macrophages. Cell Metab. 2014;20(4):614–25. doi: 10.1016/j.cmet.2014.08.010 25242226 PMC4192131

[203] Xu X, Grijalva A, Skowronski A, van Eijk M, Serlie MJ, Ferrante AW Jr. Obesity activates a program of lysosomal-dependent lipid metabolism in adipose tissue macrophages independently of classic activation. Cell Metab. 2013;18(6):816–30. doi: 10.1016/j.cmet.2013.11.001 24315368 PMC3939841

[204] Coats BR, Schoenfelt KQ, Barbosa-Lorenzi VC, Peris E, Cui C, Hoffman A, et al. Metabolically activated adipose tissue macrophages perform detrimental and beneficial functions during diet-induced obesity. Cell Rep. 2017;20(13):3149–61. doi: 10.1016/j.celrep.2017.08.096 28954231 PMC5646237

[205] Li C, Menoret A, Farragher C, Ouyang Z, Bonin C, Holvoet P, et al. Single cell transcriptomics based-MacSpectrum reveals novel macrophage activation signatures in diseases. JCI Insight. 2019;5(10):e126453. doi: 10.1172/jci.insight.126453 30990466 PMC6542613

[206] Emont MP, Jacobs C, Essene AL, Pant D, Tenen D, Colleluori G, et al. A single-cell atlas of human and mouse white adipose tissue. Nature. 2022;603(7903):926–33. doi: 10.1038/s41586-022-04518-2 35296864 PMC9504827

[207] Wentworth JM, Naselli G, Brown WA, Doyle L, Phipson B, Smyth GK, et al. Pro-inflammatory CD11c^+^CD206^+^ adipose tissue macrophages are associated with insulin resistance in human obesity. Diabetes. 2010;59(7):1648–56. doi: 10.2337/db09-0287 20357360 PMC2889764

[208] Ackermann J, Arndt L, Kirstein M, Hobusch C, Brinker G, Klöting N, et al. Myeloid cell-specific IL-4 receptor knockout partially protects from adipose tissue inflammation. J Immunol. 2021;207(12):3081–9. doi: 10.4049/jimmunol.2100699 34789558

[209] Muir LA, Cho KW, Geletka LM, Baker NA, Flesher CG, Ehlers AP, et al. Human CD206+ macrophages associate with diabetes and adipose tissue lymphoid clusters. JCI Insight. 2022;7(3):e146563. doi: 10.1172/jci.insight.146563 34990410 PMC8855803

[210] Cunha E Rocha K, Ying W, Olefsky JM. Exosome-mediated impact on systemic metabolism. Annu Rev Physiol. 2024;86:225–53. doi: 10.1146/annurev-physiol-042222-024535 38345906

[211] Mack M, Kleinschmidt A, Brühl H, Klier C, Nelson PJ, Cihak J, et al. Transfer of the chemokine receptor CCR5 between cells by membrane-derived microparticles: a mechanism for cellular human immunodeficiency virus 1 infection. Nat Med. 2000;6(7):769–75. doi: 10.1038/77498 10888925

[212] Sun N-N, Zhang Y, Huang W-H, Zheng B-J, Jin S-Y, Li X, et al. Macrophage exosomes transfer angiotensin II type 1 receptor to lung fibroblasts mediating bleomycin-induced pulmonary fibrosis. Chin Med J (Engl). 2021;134(18):2175–85. doi: 10.1097/CM9.0000000000001605 34483252 PMC8478379

[213] Borges FT, Melo SA, Özdemir BC, Kato N, Revuelta I, Miller CA, et al. TGF-β1-containing exosomes from injured epithelial cells activate fibroblasts to initiate tissue regenerative responses and fibrosis. J Am Soc Nephrol. 2013;24(3):385–92. doi: 10.1681/ASN.2012101031 23274427 PMC3582210

[214] Crewe C, Funcke J-B, Li S, Joffin N, Gliniak CM, Ghaben AL, et al. Extracellular vesicle-based interorgan transport of mitochondria from energetically stressed adipocytes. Cell Metab. 2021;33(9):1853-1868.e11. doi: 10.1016/j.cmet.2021.08.002 34418352 PMC8429176

[215] Thomou T, Mori MA, Dreyfuss JM, Konishi M, Sakaguchi M, Wolfrum C, et al. Adipose-derived circulating miRNAs regulate gene expression in other tissues. Nature. 2017;542(7642):450–5. doi: 10.1038/nature21365 28199304 PMC5330251

[216] Ying W, Gao H, Dos Reis FCG, Bandyopadhyay G, Ofrecio JM, Luo Z, et al. MiR-690, an exosomal-derived miRNA from M2-polarized macrophages, improves insulin sensitivity in obese mice. Cell Metab. 2021;33(4):781-790.e5. doi: 10.1016/j.cmet.2020.12.019 33450179 PMC8035248

[217] Rohm TV, Castellani Gomes Dos Reis F, Isaac R, Murphy C, Cunha E Rocha K, Bandyopadhyay G, et al. Adipose tissue macrophages secrete small extracellular vesicles that mediate rosiglitazone-induced insulin sensitization. Nat Metab. 2024;6(5):880–98. doi: 10.1038/s42255-024-01023-w 38605183 PMC11430498

[218] Xiong H, Liu W, Song J, Gu X, Luo S, Lu Z, et al. Adipose tissue macrophage-derived exosomal miR-210-5p in modulating insulin sensitivity in rats born small for gestational age with catch-up growth. Transl Pediatr. 2023;12(4):587–99. doi: 10.21037/tp-23-142 37181031 PMC10167396

[219] Wang H, Li H, Jiang X, Shi W, Shen Z, Li M. Hepcidin is directly regulated by insulin and plays an important role in iron overload in streptozotocin-induced diabetic rats. Diabetes. 2014;63(5):1506–18. doi: 10.2337/db13-1195 24379355

[220] Gabrielsen JS, Gao Y, Simcox JA, Huang J, Thorup D, Jones D, et al. Adipocyte iron regulates adiponectin and insulin sensitivity. J Clin Invest. 2012;122(10):3529–40. doi: 10.1172/JCI44421 22996660 PMC3461897

[221] Zhang Z, Funcke J-B, Zi Z, Zhao S, Straub LG, Zhu Y, et al. Adipocyte iron levels impinge on a fat-gut crosstalk to regulate intestinal lipid absorption and mediate protection from obesity. Cell Metab. 2021;33(8):1624-1639.e9. doi: 10.1016/j.cmet.2021.06.001 34174197 PMC8338877

[222] Joffin N, Gliniak CM, Funcke J-B, Paschoal VA, Crewe C, Chen S, et al. Adipose tissue macrophages exert systemic metabolic control by manipulating local iron concentrations. Nat Metab. 2022;4(11):1474–94. doi: 10.1038/s42255-022-00664-z 36329217 PMC11750126

[223] Ameka MK, Beavers WN, Shaver CM, Ware LB, Kerchberger VE, Schoenfelt KQ, et al. An iron refractory phenotype in obese adipose tissue macrophages leads to adipocyte iron overload. Int J Mol Sci. 2022;23(13):7417. doi: 10.3390/ijms23137417 35806422 PMC9267114

[224] Spalding KL, Arner E, Westermark PO, Bernard S, Buchholz BA, Bergmann O, et al. Dynamics of fat cell turnover in humans. Nature. 2008;453(7196):783–7. doi: 10.1038/nature06902 18454136

[225] Vandanmagsar B, Youm Y-H, Ravussin A, Galgani JE, Stadler K, Mynatt RL, et al. The NLRP3 inflammasome instigates obesity-induced inflammation and insulin resistance. Nat Med. 2011;17(2):179–88. doi: 10.1038/nm.2279 21217695 PMC3076025

[226] Haka AS, Barbosa-Lorenzi VC, Lee HJ, Falcone DJ, Hudis CA, Dannenberg AJ, et al. Exocytosis of macrophage lysosomes leads to digestion of apoptotic adipocytes and foam cell formation. J Lipid Res. 2016;57(6):980–92. doi: 10.1194/jlr.M064089 27044658 PMC4878183

[227] Flaherty SE 3rd, Grijalva A, Xu X, Ables E, Nomani A, Ferrante AW Jr. A lipase-independent pathway of lipid release and immune modulation by adipocytes. Science. 2019;363(6430):989–93. doi: 10.1126/science.aaw2586 30819964 PMC6579605

[228] Kim J, Kim SH, Kang H, Lee S, Park S-Y, Cho Y, et al. TFEB-GDF15 axis protects against obesity and insulin resistance as a lysosomal stress response. Nat Metab. 2021;3(3):410–27. doi: 10.1038/s42255-021-00368-w 33758420

[229] Odegaard JI, Ricardo-Gonzalez RR, Goforth MH, Morel CR, Subramanian V, Mukundan L, et al. Macrophage-specific PPARgamma controls alternative activation and improves insulin resistance. Nature. 2007;447(7148):1116–20. doi: 10.1038/nature05894 17515919 PMC2587297

[230] Liu C, Li P, Li H, Wang S, Ding L, Wang H, et al. TREM2 regulates obesity-induced insulin resistance via adipose tissue remodeling in mice of high-fat feeding. J Transl Med. 2019;17(1):300. doi: 10.1186/s12967-019-2050-9 31477129 PMC6720981

[231] Winn NC, Wolf EM, Garcia JN, Hasty AH. Exon 2-mediated deletion of Trem2 does not worsen metabolic function in diet-induced obese mice. J Physiol. 2022;600(20):4485–501. doi: 10.1113/JP283684 36044273 PMC9588740

[232] Sharif O, Brunner JS, Korosec A, Martins R, Jais A, Snijder B, et al. Beneficial metabolic effects of TREM2 in obesity are uncoupled from its expression on macrophages. Diabetes. 2021;70(9):2042–57. doi: 10.2337/db20-0572 33627323 PMC8576425

[233] Choi C, Jeong YL, Park K-M, Kim M, Kim S, Jo H, et al. TM4SF19-mediated control of lysosomal activity in macrophages contributes to obesity-induced inflammation and metabolic dysfunction. Nat Commun. 2024;15(1):2779. doi: 10.1038/s41467-024-47108-8 38555350 PMC10981689

[234] Grijalva A, Xu X, Ferrante AW Jr. Autophagy is dispensable for macrophage-mediated lipid homeostasis in adipose tissue. Diabetes. 2016;65(4):967–80. doi: 10.2337/db15-1219 26868294 PMC4806658

[235] Kang Y-H, Cho M-H, Kim J-Y, Kwon M-S, Peak J-J, Kang S-W, et al. Impaired macrophage autophagy induces systemic insulin resistance in obesity. Oncotarget. 2016;7(24):35577–91. doi: 10.18632/oncotarget.9590 27229537 PMC5094946

[236] Dib L, Koneva LA, Edsfeldt A, Zurke Y-X, Sun J, Nitulescu M, et al. Lipid-associated macrophages transition to an inflammatory state in human atherosclerosis increasing the risk of cerebrovascular complications. Nat Cardiovasc Res. 2023;2(7):656–72. doi: 10.1038/s44161-023-00295-x 38362263 PMC7615632

[237] Meng X, Qian X, Ding X, Wang W, Yin X, Zhuang G, et al. Eosinophils regulate intra-adipose axonal plasticity. Proc Natl Acad Sci U S A. 2022;119(3):e2112281119. doi: 10.1073/pnas.2112281119 35042776 PMC8784130

[238] Pirzgalska RM, Seixas E, Seidman JS, Link VM, Sánchez NM, Mahú I, et al. Sympathetic neuron-associated macrophages contribute to obesity by importing and metabolizing norepinephrine. Nat Med. 2017;23(11):1309–18. doi: 10.1038/nm.4422 29035364 PMC7104364

[239] Camell CD, Sander J, Spadaro O, Lee A, Nguyen KY, Wing A, et al. Inflammasome-driven catecholamine catabolism in macrophages blunts lipolysis during ageing. Nature. 2017;550(7674):119–23. doi: 10.1038/nature24022 28953873 PMC5718149

[240] Wolf Y, Boura-Halfon S, Cortese N, Haimon Z, Sar Shalom H, Kuperman Y, et al. Brown-adipose-tissue macrophages control tissue innervation and homeostatic energy expenditure. Nat Immunol. 2017;18(6):665–74. doi: 10.1038/ni.3746 28459435 PMC5438596

[241] Qiu Y, Nguyen KD, Odegaard JI, Cui X, Tian X, Locksley RM, et al. Eosinophils and type 2 cytokine signaling in macrophages orchestrate development of functional beige fat. Cell. 2014;157(6):1292–308. doi: 10.1016/j.cell.2014.03.066 24906148 PMC4129510

[242] Nguyen KD, Qiu Y, Cui X, Goh YPS, Mwangi J, David T, et al. Alternatively activated macrophages produce catecholamines to sustain adaptive thermogenesis. Nature. 2011;480(7375):104–8. doi: 10.1038/nature10653 22101429 PMC3371761

[243] Fischer K, Ruiz HH, Jhun K, Finan B, Oberlin DJ, van der Heide V, et al. Alternatively activated macrophages do not synthesize catecholamines or contribute to adipose tissue adaptive thermogenesis. Nat Med. 2017;23(5):623–30. doi: 10.1038/nm.4316 28414329 PMC5420449

[244] Giordano A, Song CK, Bowers RR, Ehlen JC, Frontini A, Cinti S, et al. White adipose tissue lacks significant vagal innervation and immunohistochemical evidence of parasympathetic innervation. Am J Physiol Regul Integr Comp Physiol. 2006;291(5):R1243-55. doi: 10.1152/ajpregu.00679.2005 16809481

[245] Knights AJ, Liu S, Ma Y, Nudell VS, Perkey E, Sorensen MJ, et al. Acetylcholine-synthesizing macrophages in subcutaneous fat are regulated by β_2_-adrenergic signaling. EMBO J. 2021;40(24):e106061. doi: 10.15252/embj.2020106061 34459015 PMC8672283

[246] Jun H, Yu H, Gong J, Jiang J, Qiao X, Perkey E, et al. An immune-beige adipocyte communication via nicotinic acetylcholine receptor signaling. Nat Med. 2018;24(6):814–22. doi: 10.1038/s41591-018-0032-8 29785025 PMC5992032

[247] Henriques F, Bedard AH, Guilherme A, Kelly M, Chi J, Zhang P, et al. Single-cell RNA profiling reveals adipocyte to macrophage signaling sufficient to enhance thermogenesis. Cell Rep. 2020;32(5):107998. doi: 10.1016/j.celrep.2020.107998 32755590 PMC7433376

[248] Severi I, Perugini J, Ruocco C, Coppi L, Pedretti S, Di Mercurio E, et al. Activation of a non-neuronal cholinergic system in visceral white adipose tissue of obese mice and humans. Mol Metab. 2024;79:101862. doi: 10.1016/j.molmet.2023.101862 38141849 PMC10792749

[249] Mottillo EP, Shen XJ, Granneman JG. β3-adrenergic receptor induction of adipocyte inflammation requires lipolytic activation of stress kinases p38 and JNK. Biochim Biophys Acta. 2010;1801(9):1048–55. doi: 10.1016/j.bbalip.2010.04.012 20435159 PMC2906652

[250] Luo S, Lin H, Wu C, Zhu L, Hua Q, Weng Y, et al. Cholinergic macrophages promote the resolution of peritoneal inflammation. Proc Natl Acad Sci U S A. 2024;121(27):e2402143121. doi: 10.1073/pnas.2402143121 38923993 PMC11228479

[251] Wang P, Loh KH, Wu M, Morgan DA, Schneeberger M, Yu X, et al. A leptin-BDNF pathway regulating sympathetic innervation of adipose tissue. Nature. 2020;583(7818):839–44. doi: 10.1038/s41586-020-2527-y 32699414

[252] Jernås M, Palming J, Sjöholm K, Jennische E, Svensson P-A, Gabrielsson BG, et al. Separation of human adipocytes by size: hypertrophic fat cells display distinct gene expression. FASEB J. 2006;20(9):1540–2. doi: 10.1096/fj.05-5678fje 16754744

[253] Li Q, Spalding KL. The regulation of adipocyte growth in white adipose tissue. Front Cell Dev Biol. 2022;10:1003219. doi: 10.3389/fcell.2022.1003219 36483678 PMC9723158

[254] Graney PL, Ben-Shaul S, Landau S, Bajpai A, Singh B, Eager J, et al. Macrophages of diverse phenotypes drive vascularization of engineered tissues. Sci Adv. 2020;6(18):eaay6391. doi: 10.1126/sciadv.aay6391 32494664 PMC7195167

[255] Shah FH, Lee H-W. Endothelial and macrophage interactions in the angiogenic niche. Cytokine Growth Factor Rev. 2024;78:64–76. doi: 10.1016/j.cytogfr.2024.07.005 39019663

[256] Cho C-H, Koh YJ, Han J, Sung H-K, Jong Lee H, Morisada T, et al. Angiogenic role of LYVE-1-positive macrophages in adipose tissue. Circ Res. 2007;100(4):e47-57. doi: 10.1161/01.RES.0000259564.92792.93 17272806

[257] Pang C, Gao Z, Yin J, Zhang J, Jia W, Ye J. Macrophage infiltration into adipose tissue may promote angiogenesis for adipose tissue remodeling in obesity. Am J Physiol Endocrinol Metab. 2008;295(2):E313-22. doi: 10.1152/ajpendo.90296.2008 18492768 PMC2519760

[258] Bourlier V, Zakaroff-Girard A, Miranville A, De Barros S, Maumus M, Sengenes C, et al. Remodeling phenotype of human subcutaneous adipose tissue macrophages. Circulation. 2008;117(6):806–15. doi: 10.1161/CIRCULATIONAHA.107.724096 18227385

[259] Hildreth AD, Ma F, Wong YY, Sun R, Pellegrini M, O’Sullivan TE. Single-cell sequencing of human white adipose tissue identifies new cell states in health and obesity. Nat Immunol. 2021;22(5):639–53. doi: 10.1038/s41590-021-00922-4 33907320 PMC8102391

[260] Rupnick MA, Panigrahy D, Zhang C-Y, Dallabrida SM, Lowell BB, Langer R, et al. Adipose tissue mass can be regulated through the vasculature. Proc Natl Acad Sci U S A. 2002;99(16):10730–5. doi: 10.1073/pnas.162349799 12149466 PMC125027

[261] Bråkenhielm E, Cao R, Gao B, Angelin B, Cannon B, Parini P, et al. Angiogenesis inhibitor, TNP-470, prevents diet-induced and genetic obesity in mice. Circ Res. 2004;94(12):1579–88. doi: 10.1161/01.RES.0000132745.76882.70 15155527

[262] Seeley RJ. Treating obesity like a tumor. Cell Metab. 2012;15(1):1–2. doi: 10.1016/j.cmet.2011.12.007 22225867

[263] Kolonin MG, Saha PK, Chan L, Pasqualini R, Arap W. Reversal of obesity by targeted ablation of adipose tissue. Nat Med. 2004;10(6):625–32. doi: 10.1038/nm1048 15133506

[264] Barnhart KF, Christianson DR, Hanley PW, Driessen WHP, Bernacky BJ, Baze WB, et al. A peptidomimetic targeting white fat causes weight loss and improved insulin resistance in obese monkeys. Sci Transl Med. 2011;3(108):108ra112. doi: 10.1126/scitranslmed.3002621 22072637 PMC3666164

[265] Gonzalez-Rellan MJ, Drucker DJ. The expanding benefits of GLP-1 medicines. Cell Rep Med. 2025;6(7):102214. doi: 10.1016/j.xcrm.2025.102214 40669447 PMC12281309

[266] Cottam MA, Itani HA, Beasley AA 4th, Hasty AH. Links between immunologic memory and metabolic cycling. J Immunol. 2018;200(11):3681–9. doi: 10.4049/jimmunol.1701713 29784764 PMC5973550

